# Risk of venous thromboembolism after SARS‐CoV‐2 vaccination—Evidence from genome‐wide association study and population‐based observational study

**DOI:** 10.1002/bcp.70614

**Published:** 2026-05-28

**Authors:** Sofia Attelind, Niclas Eriksson, Marco Cavalli, Yiyi Xu, Fredrik Nyberg, Anders Sundström, Mia Wadelius, Pär Hallberg

**Affiliations:** ^1^ Department of Medical Sciences, Clinical Pharmacogenomics Uppsala University Uppsala Sweden; ^2^ Department of Drug Safety Swedish Medical Products Agency Uppsala Sweden; ^3^ Uppsala Clinical Research Center Uppsala University Hospital Uppsala Sweden; ^4^ Clinical Immunology and Transfusion Medicine Karolinska University Hospital Stockholm Sweden; ^5^ School of Public Health and Community Medicine, Institute of Medicine, Sahlgrenska Academy University of Gothenburg Gothenburg Sweden; ^6^ Department of Pharmacy Uppsala University Uppsala Sweden

**Keywords:** Covid‐19 vaccines, drug‐related side effects and adverse reactions, genome‐wide association study, pharmacovigilance, venous thromboembolism

## Abstract

**Aim:**

We aimed to investigate whether genetic variation is associated with venous thromboembolism after immunization with SARS‐CoV‐2 vaccines.

**Methods:**

We conducted a genome‐wide association study (GWAS) on cases of venous thromboembolism within 42 days after SARS‐CoV‐2 vaccination, recruited from reports of adverse drug reactions sent to the Swedish Medical Products Agency. Two hundred one cases (43% women, 91% Swedish) were compared with 4891 Swedish population controls. Analyses were performed on two candidate variants in coagulation factor II (rs1799963) and coagulation factor V (rs6025), on 14 prespecified candidate genes and across the whole genome. To support the findings, we conducted an observational register study of the Swedish general population with/without a diagnosis of thrombophilia and the risks of venous thromboembolism after SARS‐CoV‐2 vaccination.

**Results:**

In the GWAS, the main findings were that the candidate variants of coagulation factors II rs1799963 and V rs6025 were significantly associated with venous thromboembolism (odds ratio [OR] 2.4 [95% confidence interval (CI) 1.2–4.8], *p* = .015 and OR 1.8 [95% CI 1.3–2.6], *p* = .0022). No genetic marker passed the significance threshold in the candidate gene analysis or the full genome‐wide analysis. In the register study, people with a thrombophilia diagnosis had a five‐fold elevated risk of venous thromboembolism within 42 days after vaccination, adjusted for potential confounders, OR 5.06 [95% CI 3.98–6.44].

**Conclusion:**

Well‐characterized genetic variants in the genes of coagulation factors II and V were associated with thromboembolism after SARS‐CoV‐2 immunization. Further research is recommended to elucidate their potential role in vaccine‐related thromboembolic events.

**What is already known about this subject**

- SARS‐CoV‐2 infection and some vaccines against SARS‐CoV‐2 have been associated with an increased risk of venous thromboembolism (VTE).
- It is currently unknown if genetic variation is associated with thromboembolism following SARS‐CoV‐2 vaccination.
- This is the first genome‐wide association study to investigate whether genetic variation could be associated with VTE after vaccination.

**What this study adds**

- Individuals with genetic risk variants in coagulation factor II or V were significantly more likely to develop thromboembolism after SARS‐CoV‐2 vaccination than those without these variants.
- Analyses in the general population confirm that individuals with thrombophilia diagnoses related to these variants have an increased risk of VTE after vaccination.

## 1 INTRODUCTION

The coronavirus disease 2019 (Covid‐19) pandemic, caused by severe acute respiratory syndrome coronavirus 2 (SARS‐CoV‐2), resulted in the administration of more than 980 million vaccine doses against SARS‐CoV‐2 in the European Union (EU) by the end of 2023.^1^ Several types of vaccines against SARS‐CoV‐2 were approved within the EU during the pandemic; novel messenger RNA (mRNA) vaccines, adenovirus vector vaccines and protein subunit vaccines; however, the extent to which each type was used varied between countries. In Sweden, primarily three of them were used: two mRNA vaccines (BNT162b2/Comirnaty® and mRNA‐1273/Spikevax®) and one adenovirus vector vaccine (ChAdOx1 nCoV‐19/Vaxzevria®). Sweden implemented a phased vaccination plan, initially prioritizing vaccination of those at the highest risk of Covid‐19 and in the end recommending the vaccines to all residents aged 16 years and older.

A possible causal relationship has been identified between adenovirus vector vaccines and venous thromboembolism (VTE).^2^ Vaccination with ChAdOx1 nCov‐19 has in rare cases resulted in the development of an immune thrombotic thrombocytopenia syndrome (TTS), also known as vaccine‐induced immune thrombocytopenia and thrombosis (VITT).^3^ This reaction is mediated by platelet‐activating antibodies against Platelet Factor 4 (PF4).^4^ VITT is characterized by thrombocytopenia, thrombosis (sometimes at unusual sites), elevated d‐dimer levels, reduced fibrinogen levels, sometimes bleeding and a high risk of mortality.^5^, ^6^

Thromboembolic events have also been reported for other vaccines against SARS‐CoV‐2, and the extent to which mRNA vaccines are involved has been discussed in the literature.^7^, ^8^, ^9^, ^10^ A global vaccine cohort study of 99 million vaccinated individuals^7^ showed that the observed rates of pulmonary embolism (PE) and splanchnic venous thrombosis were higher than expected for the mRNA vaccines BNT162b2 and mRNA‐1273, and the rate of cerebral venous sinus thrombosis was elevated for BNT162b2.^7^

Further, acute infection, including Covid‐19 infection, increases the risk of arterial cardiovascular events and VTE.^11^, ^12^, ^13^ The risk of VTE and PE is highest during the first 2 weeks after an infection.^11^ Other factors known to increase the risk of venous thrombosis are, for example, obesity, certain hormonal therapy, surgery, cancer and variants of the coagulation factors II (rs1799963) and V (rs6025).^14^, ^15^

In this study, we investigated whether genetic variation is associated with venous thrombotic events following Covid‐19 vaccination in a genome‐wide association study (GWAS) and further characterized the findings in an observational register study.

## 2 METHODS

### 2.1 GWAS

#### 2.1.1 Study population

Patients were recruited from nationwide spontaneous adverse drug reaction (ADR) reports sent from healthcare professionals to the Swedish Medical Products Agency (SMPA), as part of the Swedegene project.^16^ All reports from healthcare professionals of VTE associated with Covid‐19 vaccination until May 2024 were retrieved from the SMPA (Table 1). Patients were contacted and asked to participate, and those consenting were recruited. Participants had to be at least 18 years old at the time of recruitment and without a current cancer diagnosis, with a time to onset of the ADR of 1–42 days and without other strong confounding factors for thrombosis (such as surgery, infection or immobilization just before the reaction). Individuals with concomitant coagulation disorders were not excluded since this was part of what the study investigated. The time to onset of 42 days was selected based on a study of 99 million vaccinated individuals by Faksova et al.^7^

**TABLE 1:** Characteristics for patients in the genome‐wide association study with venous thromboembolism after SARS‐CoV‐2 vaccination.

	Cases (*n* = 201)
Sex (male/female)	115/86
Mean age ± SD (years [range])	63 ± 15 [18–88]
Mean BMI ± SD (kg/m^2^ [range])	28 ± 5 [18–56]
Mean time to onset ± SD (days [range])	15 ± 11 [1–41]
Ethnicity	91% Swedish origin 8% other European origin 1% other origin
Suspected drugs	BNT162b2 (Comirnaty®) *n* = 109 ChAdOx1 nCoV‐19 (Vaxzevria®) *n* = 59 mRNA‐1273 (Spikevax®) *n* = 33
Adverse drug reactions	Pulmonary embolism *n* = 125 Deep venous thrombosis *n* = 57 Thrombophlebitis *n* = 20 Thrombosis mesenteric vein, vena cava, or retinal vein *n* = 11 Cerebral venous sinus thrombosis *n* = 12
Concomitant diseases^a^	Hypertension *n* = 75 Osteoarthritis *n* = 45 Asthma *n* = 28 Depression *n* = 21 Cholecystitis/gall stones *n* = 17 Diabetes *n* = 14 Glaucoma *n* = 13 Hyperlipidaemia *n* = 12 Hypothyroidism *n* = 12
Concomitant drugs^a^	Paracetamol *n* = 56 Omeprazole *n* = 36 Budesonide inhalation *n* = 23 Losartan *n* = 19 Hydrochlorothiazide *n* = 18 Metoprolol *n* = 18 Betamethasone inhalation *n* = 17 Formoterol inhalation *n* = 17 Mometasone inhalation *n* = 17 Acetylsalicylic acid *n* = 16 Prednisolone *n* = 16 Salbutamol inhalation *n* = 16 Ibuprofen *n* = 15 Cholecalciferol *n* = 15 Levothyroxine *n* = 15 Cyanocobalamin *n* = 14 Enalapril *n* = 14 Candesartan *n* = 14 Calcium carbonate *n* = 13 Systemic hormonal replacement therapy and systemic oestrogen‐containing Contraception *n* = 12 Atorvastatin *n* = 12 Amlodipine *n* = 11 Oestradiol transdermal/vaginal *n* = 10

Abbreviation: SD = standard deviation.

^a^ Showing concomitant diseases and drugs (active substance names) that were present in at least 5% of the cohort.

Clinical data (demographics, smoking and alcohol use, medical history, concomitant drug treatment and ancestry) were collected by telephone interviews using a standardized questionnaire and by obtaining and reviewing medical records. Blood samples were drawn at the patient's nearest healthcare facility, distributed to the laboratory and stored at −70°C. All cases of VTE were adjudicated by two specialists in clinical pharmacology and drug safety to ascertain that there were no other strong confounding factors for thrombosis.

A total of 4891 unrelated individuals from the Swedish TwinGene biobank, predominantly of Swedish genetic ancestry (>99.9%) and born 1911–1958, were used as population controls.^17^

#### 2.1.2 Genome‐wide array data and quality control (QC)

DNA was extracted from peripheral venous blood. Genotyping for cases was done with the Illumina OmniExpressExome 960 K and Illumina Infinium OmniExpressExome‐8v and for controls with the Illumina Human OmniExpress 700 K array. Genotype calls were generated using the Genome Studio software from Illumina. All results are reported in accordance with the Genome Reference Consortium Human Build 38 (GRCh38).

Genome‐wide genotyping QC and data management were performed using PLINK version 1.9.^18^ QC included check of sex, and individuals were included based on per SNP call rate and individual call rate > 98%, exclusion of variants with Hardy–Weinberg equilibrium *p*‐value > 5 × 10^−8^ or minor allele frequency (MAF) < 0.5%. Following the standard QC, the data were checked *vs*. the haplotype reference consortium (HRC) data^19^ for strand issues. After the QC, the data were merged, and imputation of genotypes was performed using the TOPMED reference panel on its specific Michigan imputation server.^19^, ^20^ Post‐imputation, the data were filtered on imputation quality (minimac imputation quality *R* ^2^ > 0.7) and converted to hard calls using PLINK.^18^ The final number of variants available for analysis was 24 382 386.

Genetic principal components (PCs) were calculated using PLINK version 1.9 on the nonimputed data. In addition, PCs were estimated on the nonimputed data when merged with HapMap (release 23, 270 individuals). A scatterplot of the first two PCs is presented in the supplement with colours according to population in HapMap or Swedegene cases/controls (Figures S1–S2).

#### 2.1.3 Candidate variant and gene analyses

Candidate SNP analysis was performed on two SNPs known to be genetic risk factors for thromboembolism^21^, ^22^: the Factor II prothrombin variant rs1799963 (chromosome 11: 46739505) and the Factor V‐Leiden variant rs6025 (chromosome 1:169549811) (GRCh38).

In addition, a candidate gene analysis was performed on 14 prespecified genes (Table 2) selected from the International Society on Thrombosis and Haemostasis (ISTH) list of genes associated with coagulation and thrombosis.^23^ All individual SNPs in the candidate genes including 10 000 bases upstream/downstream were analysed separately. Further, a candidate gene set analysis, including only non‐synonymous missense variants, was performed. These variants were tagged according to dbsnp 153 [GRCh38] by using the tag maxFuncImpact id = 1583 for missense variants. Four MAF cutoffs were used in the gene set analyses: 0.01, 0.1, 0.2 and 0.4. Statistics and *p*‐values for the sequence kernel association test (SKAT), Burden and SKAT‐O tests are presented. The primary test in the gene set analyses was the SKAT‐O test, which is a combination of the SKAT and Burden tests.

**TABLE 2:** List of candidate genes involved in coagulation and thrombosis.

Gene	Ensembl ID	Chromosome	Start‐end position (GRCh38^a^)	
ADAMTS13^23^	ENSG00000160323	9	133 414 337	133 459 402
F2^23^	ENSG00000180210	11	46 740 763	46 761 056
F5^23^	ENSG00000198734	1	169 511 951	169 586 588
HRG^23^	ENSG00000113905	3	186 660 216	186 678 234
KLKB1^23^	ENSG00000164344	4	186 210 853	186 258 471
KNG1^23^	ENSG00000113889	3	186 717 348	186 744 410
PF4^4^	ENSG00000163737	4	73 980 811	73 982 124
PIGA^23^	ENSG00000165195	X	15 319 451	15 335 554
PLG^23^	ENSG00000122194	6	160 702 193	160 754 097
PROC^23^	ENSG00000115718	2	127 418 427	127 429 242
PROS1^23^	ENSG00000184500	3	93 873 051	93 980 003
SERPINC1^23^	ENSG00000117601	1	173 903 800	173 917 327
SERPIND1^23^	ENSG00000099937	22	20 774 113	20 787 720
THBD^23^	ENSG00000178726	20	23 045 633	23 049 672

^a^ Genome Reference Consortium Human Build 38.

#### 2.1.4 Statistical analyses

Power calculations for a GWAS using an additive genetic model showed that 201 cases and 4891 controls gave 80% power to detect an odds ratio (OR) of 2–3. This was based on an MAF of 10%–20% and a significance level of *p* < 5 × 10^−8^ (Figure S3). The power calculations were performed using the function GPC from the R‐package GeneticDesign.

All analyses were performed using SAIGE (Scalable and Accurate Implementation of GEneralized mixed model^24^) with Firth's bias‐reduced estimates for variants with *p*‐values below .05. SAIGE handles case–control imbalance and bias in results due to low‐frequency variants. All genome‐wide analyses were adjusted by sex and the first four genetic PCs.

SNP effects were modelled as additive. The significance level for the analyses was set according to a predefined order of hypothesis testing, where the order was candidate SNPs, candidate gene set analyses, candidate gene analyses and GWAS. The significance levels for the candidate SNP, gene set and gene analyses were set at the Bonferroni‐corrected threshold according to the number of tests in each stage. This was *p* < .05/2 = .025 for the two candidate SNPs, *p* < .05/13 = .0038 for the 13 candidate gene set analyses, as one of the selected 14 genes (PF4) did not contain any non‐synonymous missense variants, and *p* < .05/5719 = 8.7 × 10^−6^ for the candidate gene analyses including all SNPs. In the full GWAS, the conventional genome‐wide significance threshold *p* < 5 × 10^−8^ was used.

#### 2.1.5 Human leukocyte antigen (HLA) analysis

A post hoc analysis was performed to investigate HLA associations. HLA allele imputation to first and second field resolution of 181 classical HLA alleles, amino acid residues and individual SNPs was performed on the merged imputed datasets using the software SNP2HLA as implemented on the Michigan imputation server with the four‐digit multi‐ethnic HLA v1 (2021) reference panel.^25^ The threshold for significance in this analysis was adjusted to 2.76 × 10^−4^ (Bonferroni correction). HLA analyses were performed using SAIGE.

#### 2.1.6 Subgroup and sensitivity analyses

Subgroup analyses were performed for the different vaccine types: one group for the mRNA vaccines (BNT162b2 and mRNA‐1273) and another group for the adenovirus vector vaccine ChAdOx1 nCoV‐19. Furthermore, subgroup analysis was performed for the ADR PE.

In addition, a sensitivity analysis was performed excluding cases on systemic oestrogen therapy and cases with VITT.

### 2.2 Observational register study

#### 2.2.1 Data source and study population

To support the genetic analyses, a population‐based observational study was conducted to investigate whether having a diagnosis of primary thrombophilia was associated with incident VTE diagnosis within 42 days after Covid‐19 vaccination. Data were obtained from the SCIFI‐PEARL project, which links multiple national and regional registers in Sweden; details of linked registers are given elsewhere.^26^

This study included all adults who received at least one dose of any type of Covid‐19 vaccination from 27 December 2020, when vaccination became available in Sweden, to 31 August 2025 (i.e., the study period). Individuals with a cancer diagnosis from 1 January 2015 to 27 December 2020 were excluded. Individuals with any prior VTE diagnosis from 28 December 2019 to the date of their first vaccine dose were also excluded to ensure capture of incident VTE after vaccination.

#### 2.2.2 Exposure, outcome and covariates

**FIGURE 1:**
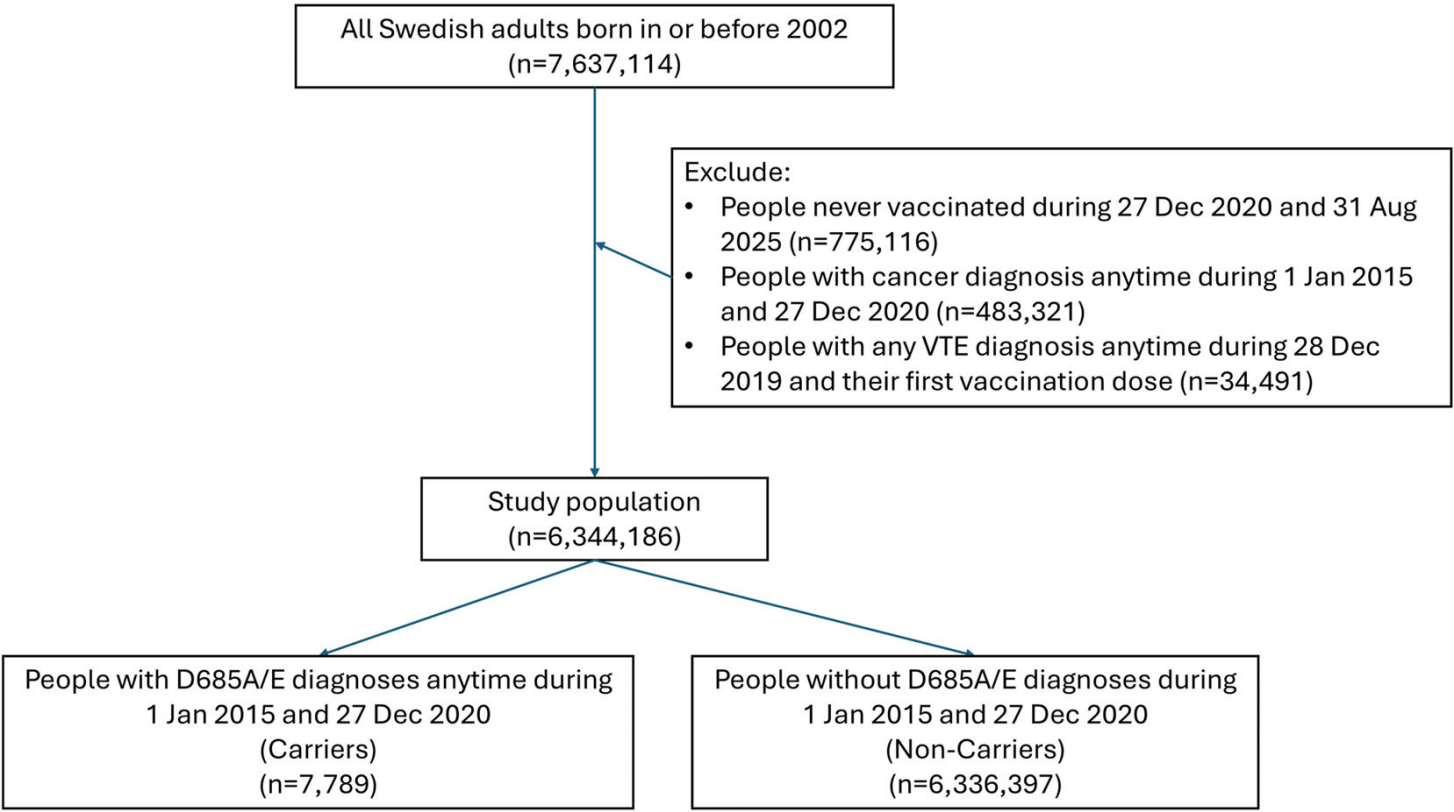
Flow chart of study population in the observational register study.

We used the Swedish version of the International Classification of Diseases, tenth revision (ICD‐10‐SE) to define both exposure and outcome. The exposure was a clinical proxy for carriers of thrombophilia‐related genetic variants, defined by the ICD‐10‐SE codes D68.5A (activated protein C resistance, factor V Leiden mutation deficiency) and/or D68.5E (prothrombin gene mutation). Individuals with these diagnoses registered at any time from 1 January 2015 to 27 December 2020 were categorized as ‘carriers’ (i.e., exposed) and individuals who did not have these diagnosis codes during the period were categorized as ‘non‐carriers’ (i.e., non‐exposed) (Figure 1).

The outcome was a clinical diagnosed VTE (either primary or secondary diagnosis from specialist outpatient visits or hospital admissions), defined by the following ICD‐10‐SE codes: I26 (pulmonary embolism), I80.1, I80.2 (deep vein thrombosis), I80.3, I80.8, I80.9, I82.1 (thrombophlebitis), I82.2 (vena cava thrombosis), H34.8 (retinal vein thrombosis) and I67.6 (cerebral venous sinus thrombosis). Only the first VTE diagnosis between the date of vaccination (index date) and 31 August 2025 for an individual was counted (i.e., no recurrence of VTE during the study period). A VTE diagnosis obtained during the 1–42 days (risk period) after any dose of Covid‐19 vaccination was considered as a positive outcome (i.e., vaccination‐related VTE), whereas no VTE diagnosis, or a VTE diagnosis outside the 42‐day window after any vaccination, was considered as a negative outcome (i.e., no vaccination‐related VTE).

The selected covariates for adjustment included age (four groups), gender (male/female), country of birth (Swedish‐born/foreign‐born), marital status (married/not married/unknown), education (primary/secondary/tertiary/unknown), income (quantiles), various relevant prior comorbidities (cardiovascular disease, hypertension, diabetes, asthma, other chronic respiratory diseases, yes/no) and Covid‐19 infection before first vaccination (yes/no). All covariates, except infection before first dose, were defined at or before the study start (27 December 2020). Details of the categories of each covariate are presented in Table 3.

**TABLE 3:** Observational register study. Baseline sociodemographic and comorbidities, as well as Covid infection status, vaccination status, and VTE among individuals from the Swedish population during the study period from 27 December 2020 to 31 August 2025.

	Non‐carrier group	Carrier group	Total
*N*	6 336 397 (99.9%)	7789 (0.1%)	6 344 186 (100.0%)
Age group			
18–25 years	685 850 (10.8%)	344 (4.4%)	686 194 (10.8%)
26–40 years	1 521 209 (24.0%)	3128 (40.2%)	1 524 337 (24.0%)
41–65 years	2 608 829 (41.2%)	2849 (36.6%)	2 611 678 (41.2%)
66–75 years	864 744 (13.6%)	838 (10.8%)	865 582 (13.6%)
≥76 years	655 765 (10.3%)	630 (8.1%)	656 395 (10.3%)
Sex			
Male	3 098 955 (48.9%)	2290 (29.4%)	3 101 245 (48.9%)
Female	3 237 442 (51.1%)	5499 (70.6%)	3 242 941 (51.1%)
Country of birth			
Sweden	5 352 655 (84.5%)	7288 (93.6%)	5 359 943 (84.5%)
Outside of Sweden	983 742 (15.5%)	501 (6.4%)	984 243 (15.5%)
Civil state			
Married	2 632 393 (41.5%)	3701 (47.5%)	2 636 094 (41.6%)
Not married	3 703 503 (58.4%)	4088 (52.5%)	3 707 591 (58.4%)
Unknown	501 (0.0%)	0 (0.0%)	501 (0.0%)
Education			
Primary	979 695 (15.5%)	824 (10.6%)	980 519 (15.5%)
Secondary	2 804 458 (44.3%)	3067 (39.4%)	2 807 525 (44.3%)
Tertiary	2 501 628 (39.5%)	3871 (49.7%)	2 505 499 (39.5%)
Unknown	50 616 (0.8%)	27 (0.3%)	50 643 (0.8%)
Household income			
Low	1 948 732 (30.8%)	2341 (30.1%)	1 951 073 (30.8%)
Medium	2 147 706 (33.9%)	2861 (36.7%)	2 150 567 (33.9%)
Unknown	2 239 458 (35.3%)	2587 (33.2%)	2 242 045 (35.3%)
Cardiovascular disease			
No	5 812 302 (91.7%)	6018 (77.3%)	5 818 320 (91.7%)
Yes	524 095 (8.3%)	1771 (22.7%)	525 866 (8.3%)
Hypertension			
No	5 706 598 (90.1%)	6517 (83.7%)	5 713 115 (90.1%)
Yes	629 799 (9.9%)	1272 (16.3%)	631 071 (9.9%)
Diabetes			
No	6 333 324 (100.0%)	7784 (99.9%)	6 341 108 (100.0%)
Yes	3073 (0.0%)	5 (0.1%)	3078 (0.0%)
Asthma			
No	6 196 667 (97.8%)	7369 (94.6%)	6 204 036 (97.8%)
Yes	139 730 (2.2%)	420 (5.4%)	140 150 (2.2%)
COPD			
No	6 269 168 (98.9%)	7645 (98.2%)	6 276 813 (98.9%)
Yes	67 229 (1.1%)	144 (1.8%)	67 373 (1.1%)
Vaccination status			
1 dose	103 872 (1.6%)	111 (1.4%)	103 983 (1.6%)
2 doses	1 288 641 (20.3%)	1369 (17.6%)	1 290 010 (20.3%)
3 doses	2 220 950 (35.1%)	2764 (35.5%)	2 223 714 (35.1%)
4 doses	844 043 (13.3%)	1238 (15.9%)	845 281 (13.3%)
≥5 doses	1 878 891 (29.7%)	2307 (29.6%)	1 881 198 (29.7%)
No infection before vaccination	5 595 949 (88.3%)	6739 (86.5%)	5 602 688 (88.3%)
Infection before vaccination	740 448 (11.7%)	1050 (13.5%)	741 498 (11.7%)
No VTE within 42 days after vaccination	6 323 745 (99.8%)	7720 (99.1%)	6 331 465 (99.8%)
VTE yes within 42 days after vaccination	12 652 (0.2%)	69 (0.9%)	12 721 (0.2%)

Abbreviations: COPD = chronic obstructive pulmonary disease; *N* = number of individuals in each group; VTE = venous thromboembolism.

#### 2.2.3 Statistical analysis

Logistic regression was used to estimate the OR and 95% confidence interval (CI) of obtaining a VTE diagnosis within 1–42 days after any vaccination between the ‘carrier’ and ‘non‐carrier’ group. ORs and 95% CIs were reported from a crude model (without any adjustments) and an adjusted model, respectively. Analyses were performed using a standard software package (Stata, version 18.0; StataCorp LLC).

#### 2.2.4 Compliance with ethical standards

This study was approved by the Swedish Ethical Review Authority (2021‐02212, as well as 2020‐01800 with amendments) and the regional ethical review board in Stockholm (2007/644‐31 and 2011/463‐32). Research was conducted according to the latest update of the Declaration of Helsinki, and all study participants in the GWAS gave written informed consent. For the population‐based register analysis, informed consent is not needed.

#### 2.2.5 Nomenclature of targets and ligands

Key protein targets and ligands in this article are hyperlinked to corresponding entries in http://www.guidetopharmacology.org and are permanently archived in the Concise Guide to PHARMACOLOGY 2023/24.^27^

## 3 RESULTS

### 3.1 Genetic analysis

#### 3.1.1 Study population

A total of 222 cases were recruited, of which 21 were excluded for the following reasons; too long time to onset (*n* = 10), insufficient medical information available (*n* = 4), vaccine not suspected to be causative (*n* = 3), did not pass genotyping QC (*n* = 2), one single case vaccinated with protein subunit vaccine NVX‐CoV2373 (Nuvaxovid®) (*n* = 1) and ongoing cancer (*n* = 1).

A total of 201 cases thus remained, and their characteristics are shown in Table 1. Most cases were vaccinated with BNT162b2 (*n* = 109), followed by ChAdOx1 nCoV‐19 (*n* = 59) and mRNA‐1273 (*n* = 33). The majority, 57%, were male, and 91% were of Swedish origin. The ADRs were PE (*n* = 125) including three VITT cases, deep venous thrombosis (*n* = 57) including one VITT case, thrombophlebitis (*n* = 20), thrombosis mesenteric vein, vena cava, or retinal vein (*n* = 11), and cerebral venous sinus thrombosis (*n* = 12).

A total of 12 cases on systemic oestrogen therapy and four with VITT were excluded in the sensitivity analysis.

A plot of the first two genetic PCs for all cases of thromboembolism (*n* = 201) and all controls (*n* = 4891) is shown in Figure S1. A plot of PC1 and PC2 for all cases of thromboembolism and all controls is also presented in Figure S2 after merging the data with HapMap. There were no major deviations in cases and controls from the European cluster.

#### 3.1.2 Candidate variant analyses

**FIGURE 2:**
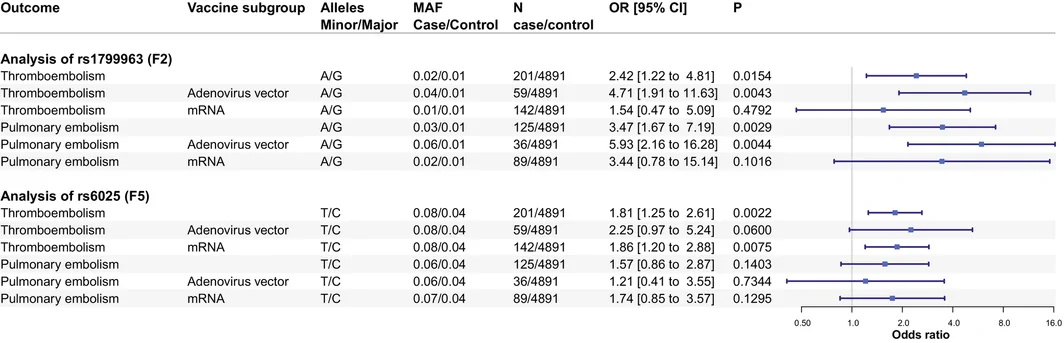
Forest plot for rs1799963 (Factor II) and rs6025 (Factor V) in the genome‐wide association study analyses of thromboembolism in the imputed data. Adenovirus vector = ChAdOx1 nCoV‐19/Vaxzevria®; CI = confidence interval; F2 = Factor 2; F5 = Factor V; MAF = minor allele frequency; mRNA = BNT162b2/Comirnaty® and mRNA‐1273/Spikevax®; *N* = number of cases and controls; OR = odds ratio.

The candidate SNP rs1799963 was significantly associated with VTE (OR 2.4 [95% CI] 1.2–4.8], *p* = .015) in the full analysis (*n* = 201) (Figure 2). This was also the case for PE. The effect appeared to be strongest for ChAdOx1 nCoV‐19, but confidence intervals were large and overlapping, and this observation is thus uncertain.

The candidate SNP rs6025 was significantly associated with VTE (OR 1.8 [95% CI 1.3–2.6], *p* = .0022) in the full analysis (*n* = 201) (Figure 2). There were no clear differences between vaccine types.

The sensitivity analysis, excluding cases on systemic oestrogen therapy and VITT, showed that the results remained unchanged (Figure S4).

#### 3.1.3 Candidate gene analyses

The candidate gene set analyses and candidate gene analyses revealed no statistically significant associations with VTE (Tables S1–S2). The sensitivity analysis, excluding cases of systemic oestrogen therapy and VITT, did not change this result.

#### 3.1.4 Genome‐wide association analyses

**FIGURE 3:**
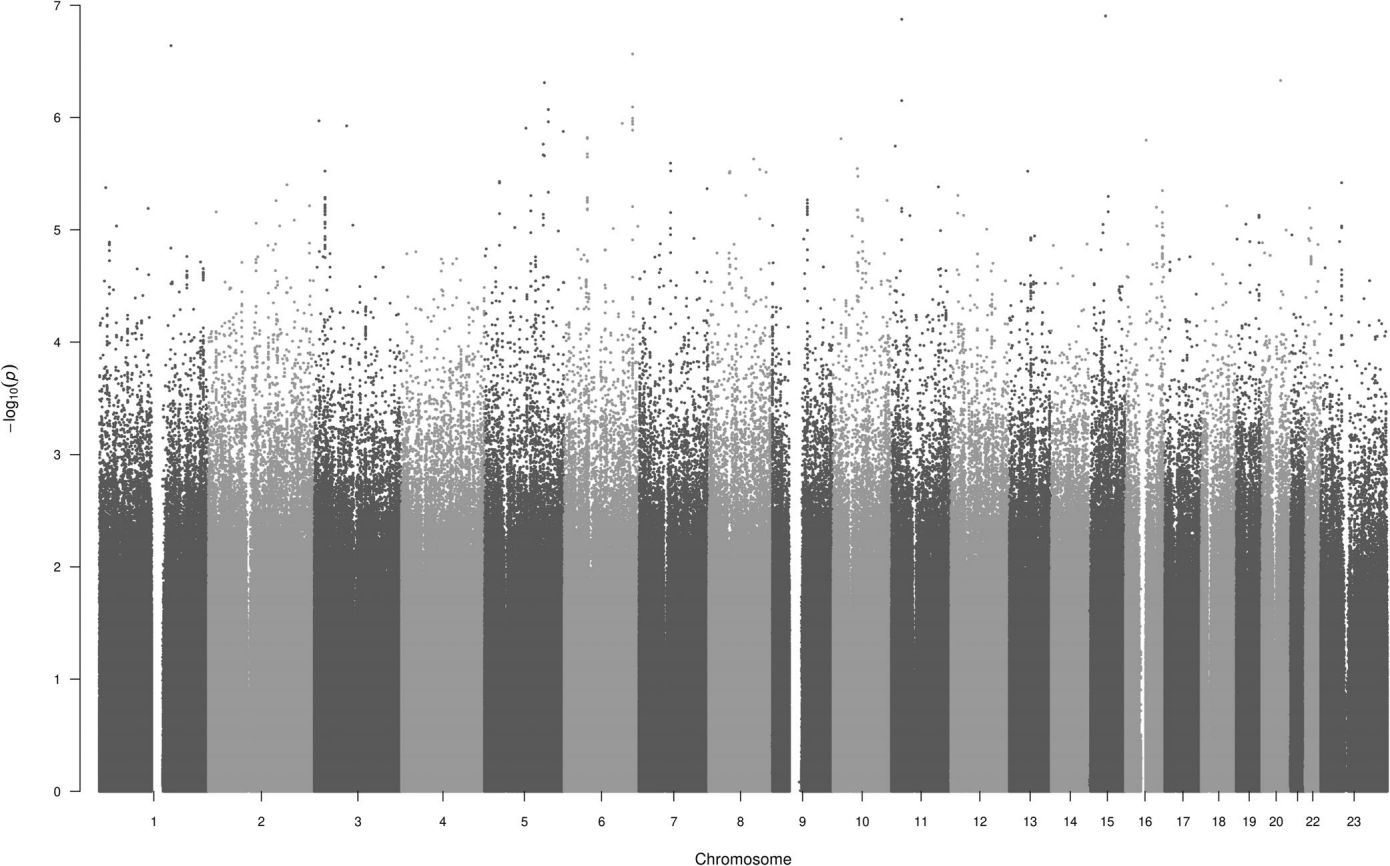
Manhattan plot for the full genome‐wide association study analysis of thromboembolism following SARS‐CoV‐2 vaccination (201 cases *vs.* 4891 population controls), adjusted by sex and the first four genetic principal components. No marker reached the threshold for statistical significance (*p* < 5 × 10^−8^).

No genetic marker passed the significance threshold in the full genome‐wide analysis (*n* = 201) (Figure 3 and Table S3). In the sensitivity analysis (*n* = 185), a few genetic markers passed the significance threshold; however, their location suggested no clinical consequence. For example, the SNP rs185809943 on chromosome 15, in the long non‐coding RNA gene *TCF12‐DT*, passed the significance threshold in the full GWAS sensitivity analysis (*n* = 185) (Figure S5; OR 8.5 [95% CI 4.5–16.2], *p* = 4.9 × 10^−8^). Also, in the analysis restricted to only SARS‐CoV‐2 mRNA vaccines (*n* = 130), SNPs in or nearby the non‐coding RNA gene *LOC105379110* on chromosome 5 (top hit rs1272761321, OR 8.3 [95% CI 4.5–15.1], *p* = 1.9 × 10^−8^) were significantly associated with thromboembolism (Figure S6).

In addition, rs561929057, on chromosome 12, was significantly associated with thromboembolism in the analysis restricted to only the adenovirus vector vaccine (ChAdOx1 nCoV‐19, *n* = 55) (OR 175.1 [95% CI 32.0–956.4], *p* = 4.05 × 10^−8^). This is a rare non‐coding variant (Figure S7).

#### 3.1.5 HLA analysis

There were no statistically significant findings in the analysis of HLA associations with VTE (Table S4).

### 3.2 Observational register study

Among the study population, 7789 individuals (0.1%) were in the carrier group. Compared with non‐carriers, the carrier group was more often female (70.9% *vs*. 50.4%), of working age 26–65 years (76.8% *vs*. 65.2%) and more frequently born in Sweden. Comorbidity burden was higher in the carrier group, with cardiovascular disease, hypertension, asthma and chronic obstructive pulmonary disease (COPD) all more prevalent. Vaccination was quite similar across the two groups. Prior infection before the first vaccination dose was slightly more common in the carrier group. VTE within 42 days after vaccination was in general a rare event; however, the carrier group was more often diagnosed with VTE shortly after vaccination compared to the non‐carrier group (Table 3).

The carrier group showed more than four times higher odds of a VTE diagnosis within 42 days after any dose of Covid‐19 vaccination (OR 4.47 [95% CI 3.52–5.67]; Table 4). After adjusting for potential confounders, the OR was 5.06 [95% CI 3.98–6.44] (Table 4).

**TABLE 4:** Observational register study. Odds ratio (OR) and 95% confidence interval (CI) of obtaining a VTE diagnosis within 42 days after vaccination among the carrier group compared with the non‐carrier group.

	Raw model		Adjusted model	
	OR (95% CI)	*p*	OR (95% CI)	*p*
Non‐carrier group	Ref		Ref	
Carrier group	4.47 (3.52, 5.67)	<.001	5.06 (3.98, 6.44)	<.001

Abbreviation: VTE = venous thromboembolism.

## 4 DISCUSSION

This study aimed to investigate whether genetic variation is associated with VTE after SARS‐CoV‐2 vaccination through two study setting approaches, the GWAS and the population‐based observational study. The results of the GWAS indicate an association with the well‐known variants rs1799963 in coagulation factor II and rs6025 in coagulation factor V and an increased risk of thromboembolism after vaccination. Such an association was further supported by the observational study, showing that people with clinical diagnosis of thrombophilia had higher odds of receiving a VTE diagnosis within 42 days after any dose of vaccination. However, we cannot conclusively say from this study that the SARS‐CoV‐2 vaccination in itself increases the risk, as it may instead be related to the underlying condition and/or overall higher background risk of VTE among the carriers of the genetic variation.

VTE is a multifactorial disease,^28^ and several risk factors are involved in the pathogenesis. It is nevertheless interesting that known genetic risk factors for thromboembolism were associated with an increased risk of thromboembolism after SARS‐CoV‐2 vaccination. An association with specific inherited thrombophilias, including activated protein C (APC) resistance caused by the F5 variant rs6025, and the risk of developing thromboembolism has been reported in patients with Covid‐19 infection, contributing to higher disease‐related mortality.^29^ Thus, immunization with SARS‐CoV‐2 vaccines is beneficial also for this risk group but would suggest proper monitoring, that is, alertness for symptoms of VTE and prompt anticoagulant therapy if needed.

The genetic markers that passed the significance threshold in the GWAS analyses in the sensitivity analysis subset are of doubtful clinical relevance. For example, VTE after SARS‐CoV‐2 mRNA vaccination was associated with the *LOC105379110* gene, which is a non‐coding RNA gene with no known relation to coagulation. This association may thus have been a spurious finding.

The results from the observational register study were consistent with the conclusion from the GWAS analysis, that is, people with a clinical proxy for carriage of thrombophilia‐related genetic variation had a higher likelihood of developing VTE shortly after Covid‐19 vaccination. However, as already noted, these findings cannot disentangle whether the increased odds of receiving a VTE diagnosis after vaccination are due to the group's higher baseline VTE risk or to an effect of the vaccination itself. Therefore, the current results should not be interpreted as scientific evidence against recommending Covid‐19 vaccination for individuals with a clinical proxy for carriers of thrombophilia‐related genetic variants. These results are in accordance with the results of a study by Xie et al.^30^ investigating a polygenic risk score including rs1799963 (Factor II) and rs6025 (Factor V) in the UK Biobank, demonstrating that at the population level, VTE occurring after vaccination showed a genetic aetiology similar to that of conventional VTE.

The study has certain limitations. We were only able to include 201 patients in the GWAS, and the power to detect associated variants was thus limited. In addition, almost all participants were of white European ancestry, and therefore, the results cannot be generalized to other ancestries. The observational study was based on register data from the Swedish National Patient Register (NPR), which has demonstrated high diagnostic accuracy for VTE, with a positive predictive value of 95%,^31^ making substantial misclassification of VTE unlikely. However, the number of registered ICD codes for coagulation factor II and V risk variants was lower than expected based on their prevalence in the Swedish population.^32^ This discrepancy reflects the fact that the NPR captures only individuals who have sought healthcare and received a corresponding diagnosis, rather than all carriers in the general population. Any such misclassification would be expected to bias the results towards the null.

## 5 CONCLUSION

To our knowledge, this is the first GWAS to investigate whether genetic variation is associated with VTE after SARS‐CoV‐2 vaccination. The variants of coagulation factors II rs1799963 and V rs6025 were associated with thromboembolism after SARS‐CoV‐2 vaccination. An increased risk for VTE among carriers of thrombophilia‐related genetic variation was also supported in an observational register study. Additional research is required to elucidate their potential role in vaccine‐related thromboembolic events.

## AUTHOR CONTRIBUTIONS

All authors contributed to the conceptualization of this work and the design of the study. S.A. and P.H. adjudicated the cases. N.E. performed the GWAS data analysis and in the observational register study. Y.X. performed the data analysis. S.A. drafted the first version of the manuscript, and all authors interpreted the results. All authors read, revised, edited and approved the final version of the manuscript.

## CONFLICT OF INTEREST STATEMENT

S.A. is employed by the Swedish Medical Products Agency, Uppsala, Sweden. The views expressed in this paper are the personal views of the authors and not necessarily the views of the Government agency. F.N. reports participation in research projects funded by Bayer and AstraZeneca (regulator‐mandated phase IV study; investigator‐initiated study), with funds paid to the University of Gothenburg, where he is employed (no personal fees), with no relation to the work reported here. F.N. holds some AstraZeneca shares. N.E., M.C., Y.X., A.S., M.W. and P.H. declare that they have no conflicts of interest.

## Supporting information


**Figure S1.**Principal component (PC) plot of PC1 and PC2 for all cases of thromboembolism (*n* = 201) and all controls (*n* = 4891).
**Figure S2.** Principal component (PC) plot of PC1 and PC2 for all cases of thromboembolism (*n* = 201) and all controls (*n* = 4891). Comparison in HapMap.
**Figure S3.** Power calculation for genome‐wide association study using an additive genetic model with 201 cases and 4891 controls, 1 million tests and assumed disease prevalence of 0.002.
**Figure S4.** Forest plot for rs6025 (Factor V) and rs1799963 (Factor II) in the genome‐wide association study analyses of thromboembolism in the imputed data including the sensitivity analyses.
**Figure S5.** Manhattan plot of the genome‐wide association study sensitivity analysis of thromboembolism in the imputed data (*n* = 185).
**Figure S6.** Manhattan plot of the mRNA subgroup genome‐wide association study sensitivity analysis of thromboembolism in the imputed data (*n* = 130).
**Figure S7.** Manhattan plot of the adenovirus vector vaccine subgroup genome‐wide association study sensitivity analysis of thromboembolism in the imputed data (*n* = 55).
**Table S1.** Candidate gene results for the gene set analyses for all cases of thromboembolism (*n* = 201) *vs.* all controls (*n* = 4891).
**Table S2.** Top results for the candidate gene regions from the genome‐wide association study analyses of the outcome thromboembolism (*n* = 201) *vs.* all population controls (*n* = 4891).
**Table S3.** Top 30 results of the genome‐wide association analysis for all cases of thromboembolism (*n* = 201) *vs.* all controls (*n* = 4891).
**Table S4.** Post hoc: Human leukocyte antigen (HLA) imputation results. Top results for the HLA analyses of the outcome thromboembolism (*n* = 201) *vs.* all population controls (*n* = 4891).
**Table S5.** Top results of the genome‐wide association sensitivity analysis for cases of thromboembolism (*n* = 185) *vs.* all controls (*n* = 4891).
**Table S6.** Top results of the mRNA subgroup genome‐wide association sensitivity analysis for cases of thromboembolism (*n* = 130) *vs.* all controls (*n* = 4891).
**Table S7.** Top results of the adenovirus vector vaccine subgroup genome‐wide association sensitivity analysis for cases of thromboembolism (*n* = 55) *vs.* all controls (*n* = 4891).

## Data Availability

The datasets generated and analysed in the GWAS are not publicly available due to the European General Data Protection Regulation (GDPR), which requires us to protect the identity of participants, but datasets are partly available from the corresponding author on reasonable request. The register study used pseudonymized individual‐level data from Swedish healthcare registers that are not publicly available according to Swedish legislation. The data can be obtained from the respective Swedish data holders on the basis of ethics approval for the research in question, subject to relevant legislation, processes and data protection.
