# Supplementary material for: Risk of venous thromboembolism after SARS‐CoV‐2 vaccination—Evidence from genome‐wide association study and population‐based observational study
Source: Br J Clin Pharmacol. 2026 May 28;92(9):3246–56. doi: 10.1002/bcp.70614 (PMC13519795; doi:10.1002/bcp.70614)
Supplement: Supplementary file 1 [file BCP-92-3246-s001.pdf]

Supporting information

Risk of venous thromboembolism after SARS-CoV-2 vaccination – evidence from genome-wide association study and population-based observational study

Sofia Attelind[1,2], Niclas Eriksson[1,3], Marco Cavalli[1,4], Yiyi Xu[5], Fredrik Nyberg[5], Anders Sundström[6], Mia Wadelius[1], and Pär Hallberg[1].

[1] Department of Medical Sciences, Clinical Pharmacogenomics, Uppsala University, Uppsala, Sweden

[2] Department of Drug Safety, Swedish Medical Products Agency, Uppsala, Sweden

[3] Uppsala Clinical Research Center, Uppsala University Hospital, Uppsala, Sweden

[4] Clinical Immunology and Transfusion Medicine, Karolinska University Hospital, Stockholm, Sweden

[5] School of Public Health and Community Medicine, Institute of Medicine, Sahlgrenska Academy, University of Gothenburg, Sweden

[6] Department of Pharmacy, Uppsala University, Uppsala, Sweden

Contents

Supporting Figure S1. Principal component (PC) plot of PC1 and PC2 for all cases of thromboembolism (n = 201) and all controls (n = 4,891)........2

Supporting Figure S2. Principal component (PC) plot of PC1 and PC2 for all cases of thromboembolism (n = 201) and all controls (n = 4,891). Comparison in HapMap........3

Supporting Figure S3. Power calculation for genome-wide association study using an additive genetic model with 201 cases and 4,891 controls, 1 million tests and assumed disease prevalence of 0.002........4

Supporting Figure S4. Forest plot for rs6025 (factor V) and rs1799963 (factor II) in the genome-wide association study analyses of thromboembolism in the imputed data including the sensitivity analyses.......5

Supporting Figure S5. Manhattan plot of the genome-wide association study sensitivity analysis of thromboembolism in the imputed data (n = 185)........5

Supporting Figure S6. Manhattan plot of the mRNA subgroup genome-wide association study sensitivity analysis of thromboembolism in the imputed data (n = 130)........6

Supporting Figure S7. Manhattan plot of the adenovirus vector vaccine subgroup genome-wide association study sensitivity analysis of thromboembolism in the imputed data (n = 55)........6

Supporting Table S1. Candidate gene results for the gene set analyses for all cases of thromboembolism (n = 201) vs all controls (n = 4,891)........7

Supporting Table S2. Top results for the candidate gene regions from the genome-wide association study analyses of the outcome thromboembolism (n = 201) vs all population controls (n = 4,891)........9

Supporting Table S3. Top 30 results of the genome-wide association analysis for all cases of thromboembolism (n=201) vs all controls (n=4,891)........12

Supporting Table S4. Post-hoc: Human Leukocyte Antigen (HLA) imputation results. Top results for the HLA analyses of the outcome thromboembolism (n = 201) vs all population controls (n = 4,891)........14

Supporting Table S5. Top results of the genome-wide association sensitivity analysis for cases of thromboembolism (n=185) vs all controls (n=4,891)........17

Supporting Table S6. Top results of the mRNA subgroup genome-wide association sensitivity analysis for cases of thromboembolism (n=130) vs all controls (n=4,891)........18

Supporting Table S7. Top results of the adenovirus vector vaccine subgroup genome-wide association sensitivity analysis for cases of thromboembolism (n=55) vs all controls (n=4,891)........19

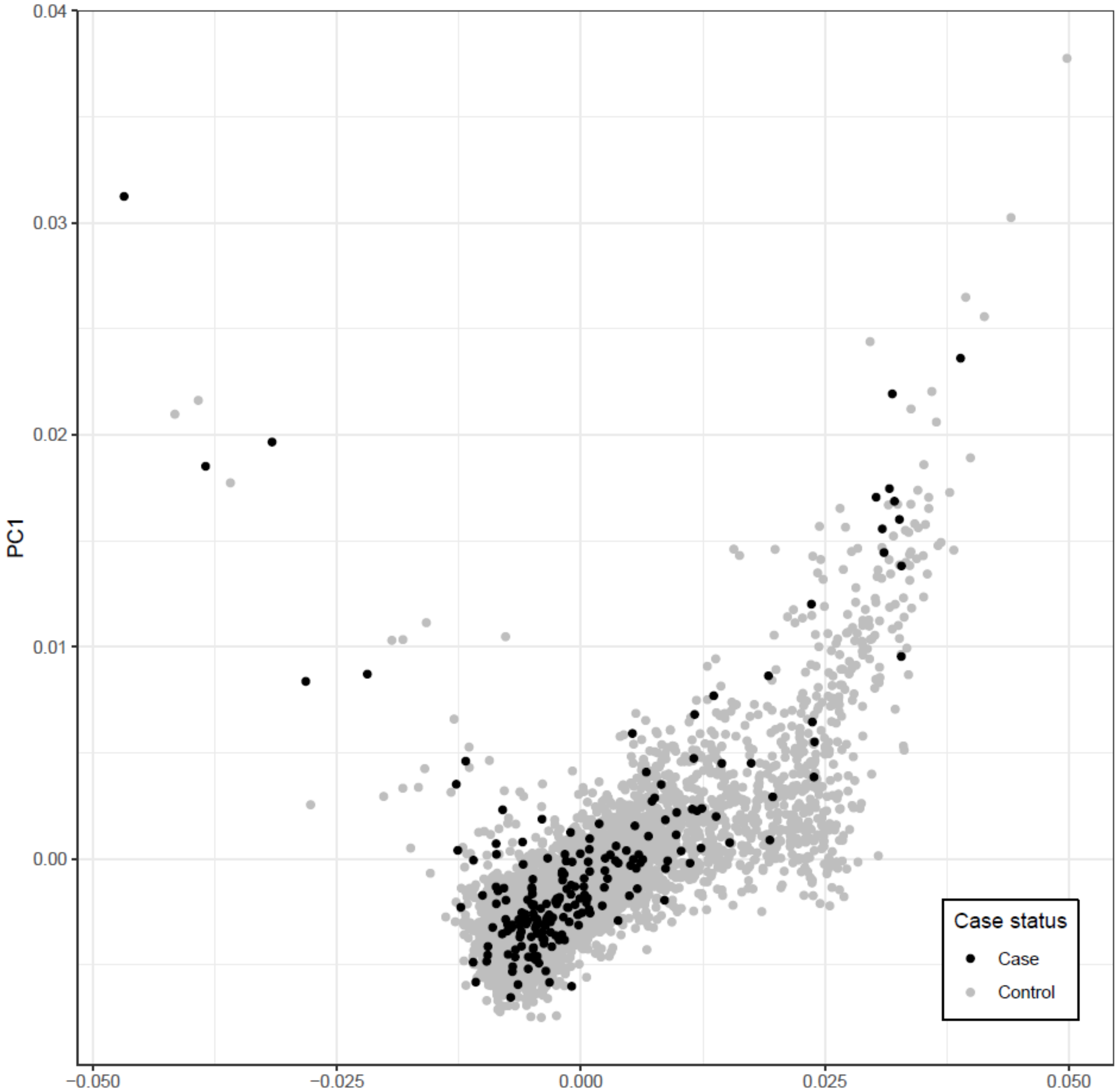

Supporting Figure S1. Principal component (PC) plot of PC1 and PC2 for all cases of thromboembolism (n = 201) and all controls (n = 4,891).

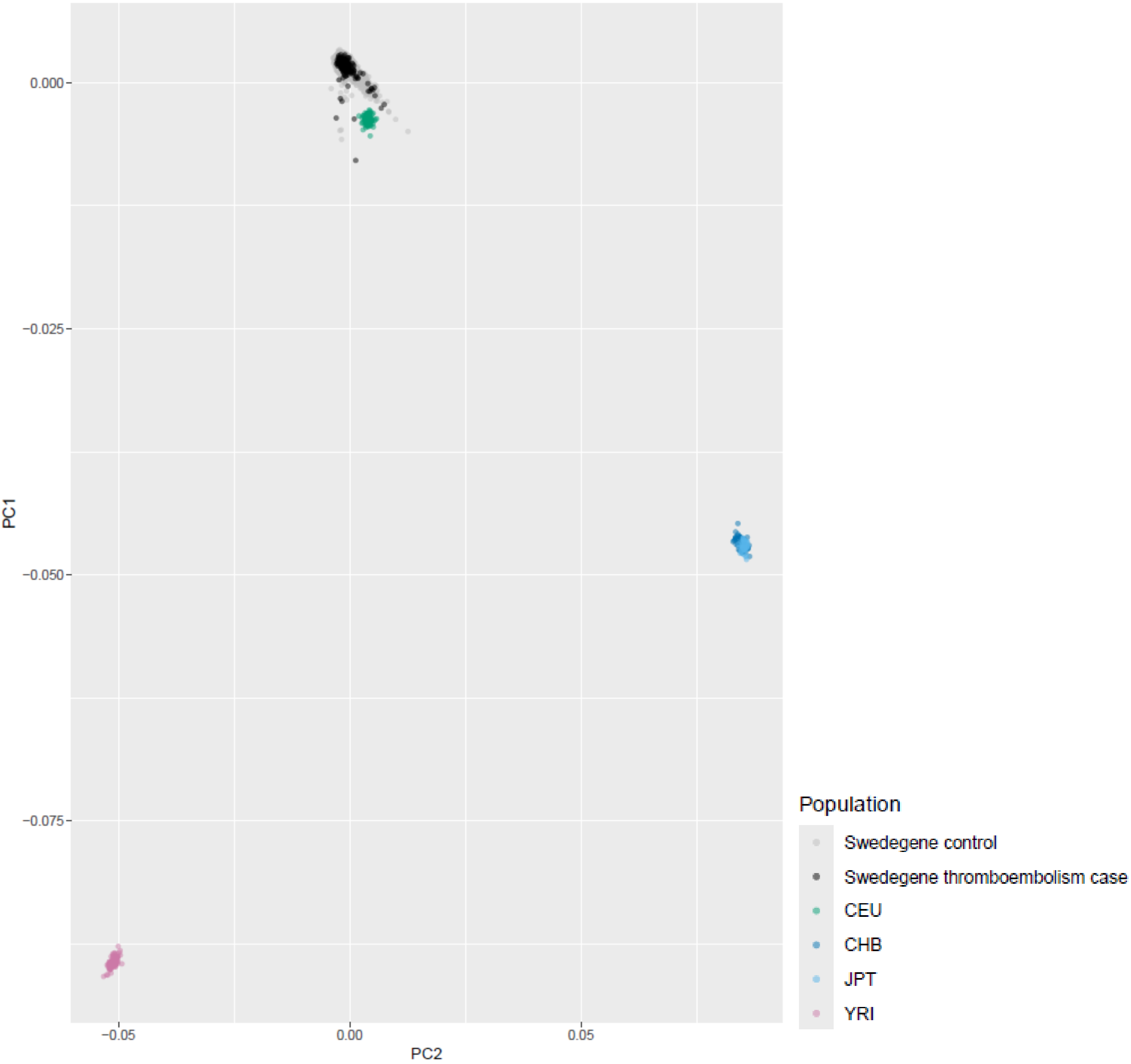

Supporting Figure S2. Principal component (PC) plot of PC1 and PC2 for all cases of thromboembolism (n = 201) and all controls (n = 4,891). Comparison in HapMap with Utah residents with Northern and Western European ancestry from the CEPH collection (CEU), Han Chinese in Beijing, China (CHB), Japanese in Tokyo, Japan (JPT), and Yoruba people in Ibadan, Nigeria (YRI).

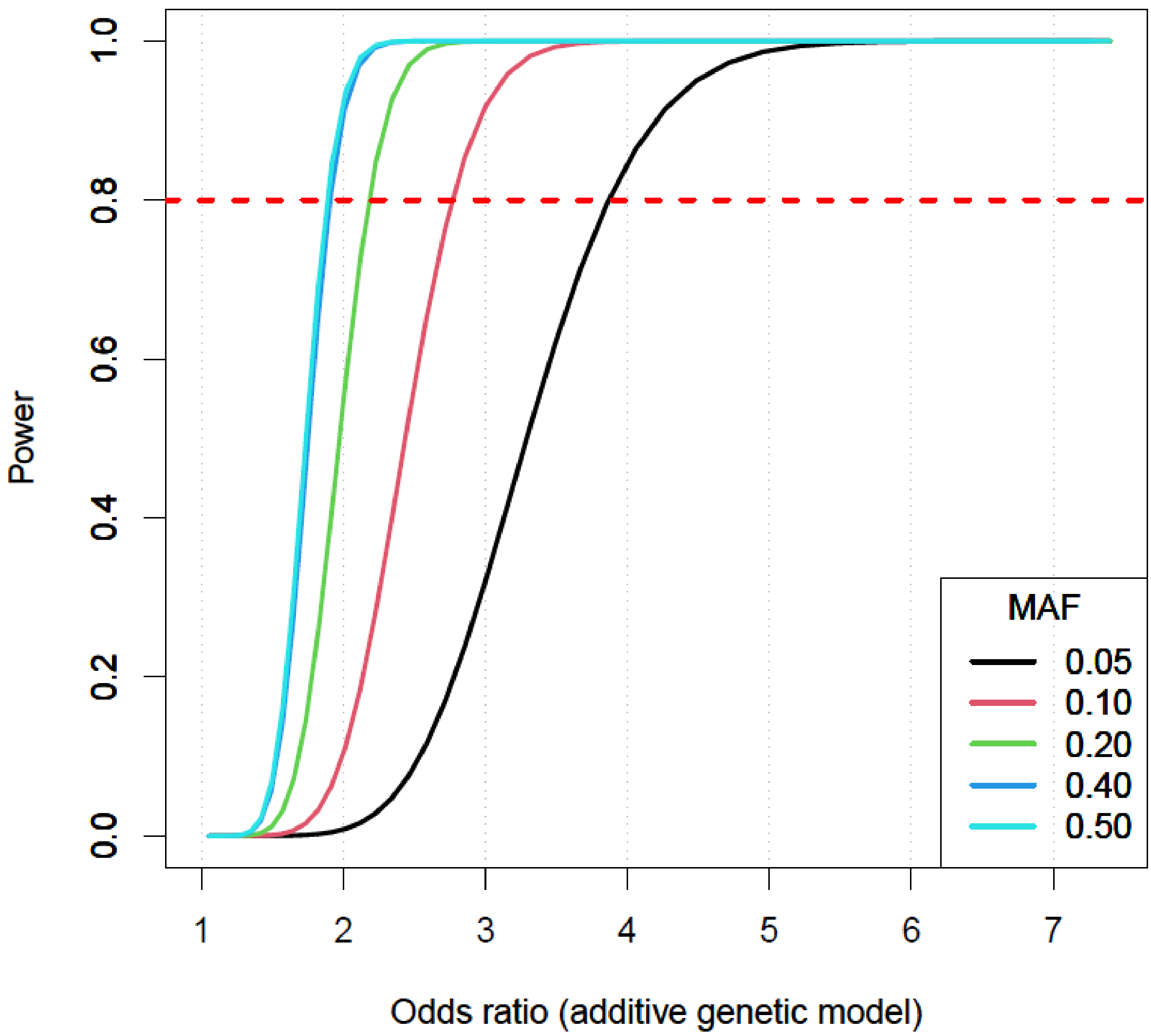

Supporting Figure S3. Power calculation for genome-wide association study using an additive genetic model with 201 cases and 4,891 controls, 1 million tests and assumed disease prevalence of 0.002.

Outcome	Vaccine subgroup	Alleles Minor/Major	MAF Case/Control	N case/control	OR [95% CI]	P
**Analysis of rs1799963 (F2)**						
Thromboembolism		A/G	0.02/0.01	201/4891	2.42 [1.22 to 4.81]	0.0154
Thromboembolism	Adenovirus vector	A/G	0.04/0.01	59/4891	4.71 [1.91 to 11.63]	0.0043
Thromboembolism	mRNA	A/G	0.01/0.01	142/4891	1.54 [0.47 to 5.09]	0.4792
Pulmonary embolism		A/G	0.03/0.01	125/4891	3.47 [1.67 to 7.19]	0.0029
Pulmonary embolism	Adenovirus vector	A/G	0.06/0.01	36/4891	5.93 [2.16 to 16.28]	0.0044
Pulmonary embolism	mRNA	A/G	0.02/0.01	89/4891	3.44 [0.78 to 15.14]	0.1016
**Analysis of rs6025 (F5)**						
Thromboembolism		T/C	0.08/0.04	201/4891	1.81 [1.25 to 2.61]	0.0022
Thromboembolism	Adenovirus vector	T/C	0.08/0.04	59/4891	2.25 [0.97 to 5.24]	0.0600
Thromboembolism	mRNA	T/C	0.08/0.04	142/4891	1.86 [1.20 to 2.88]	0.0075
Pulmonary embolism		T/C	0.06/0.04	125/4891	1.57 [0.86 to 2.87]	0.1403
Pulmonary embolism	Adenovirus vector	T/C	0.06/0.04	36/4891	1.21 [0.41 to 3.55]	0.7344
Pulmonary embolism	mRNA	T/C	0.07/0.04	89/4891	1.74 [0.85 to 3.57]	0.1295
**Sensitivity analysis of rs1799963 (F2)**						
Thromboembolism	(sens)	A/G	0.02/0.01	185/4891	2.66 [1.33 to 5.30]	0.0090
Thromboembolism	Adenovirus vector (sens)	A/G	0.05/0.01	55/4891	5.11 [2.06 to 12.65]	0.0031
Thromboembolism	mRNA (sens)	A/G	0.02/0.01	130/4891	1.76 [0.50 to 6.12]	0.3758
Pulmonary embolism	(sens)	A/G	0.03/0.01	116/4891	3.77 [1.81 to 7.84]	0.0018
Pulmonary embolism	Adenovirus vector (sens)	A/G	0.06/0.01	33/4891	6.49 [2.35 to 17.93]	0.0032
Pulmonary embolism	mRNA (sens)	A/G	0.02/0.01	83/4891	4.03 [0.87 to 18.61]	0.0746
**Sensitivity analysis of rs6025 (F5)**						
Thromboembolism	(sens)	T/C	0.08/0.04	185/4891	1.77 [1.21 to 2.61]	0.0047
Thromboembolism	Adenovirus vector (sens)	T/C	0.08/0.04	55/4891	2.09 [0.87 to 5.00]	0.0979
Thromboembolism	mRNA (sens)	T/C	0.08/0.04	130/4891	1.86 [1.18 to 2.94]	0.0106
Pulmonary embolism	(sens)	T/C	0.06/0.04	116/4891	1.31 [0.70 to 2.45]	0.3983
Pulmonary embolism	Adenovirus vector (sens)	T/C	0.05/0.04	33/4891	0.96 [0.31 to 2.95]	0.9413
Pulmonary embolism	mRNA (sens)	T/C	0.06/0.04	83/4891	1.48 [0.70 to 3.12]	0.3042

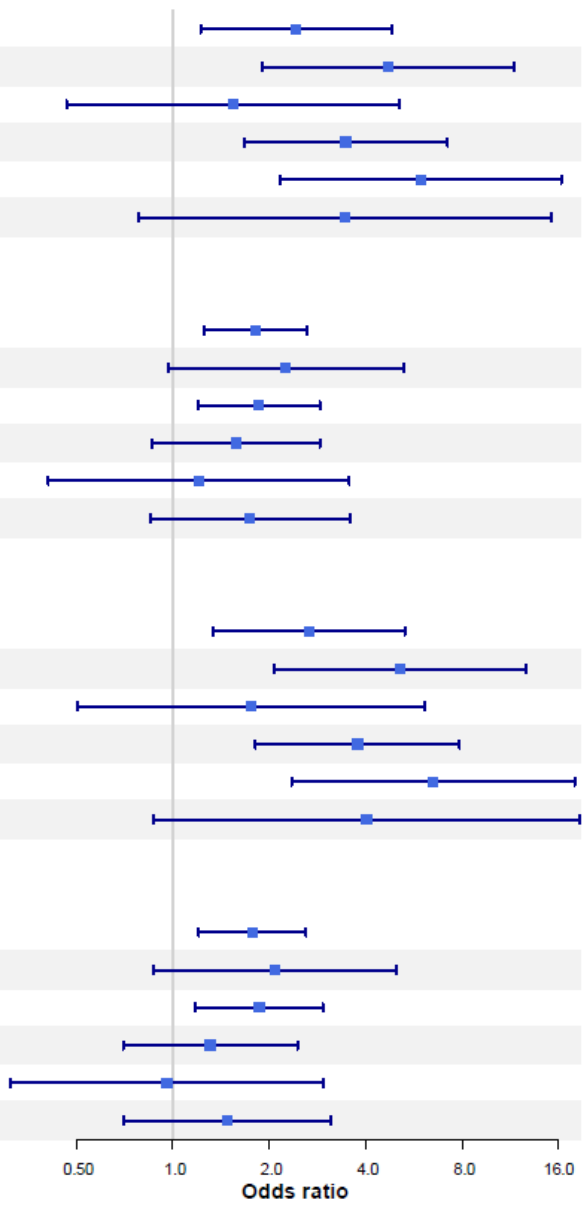

Supporting Figure S4. Forest plot for rs6025 (factor V) and rs1799963 (factor II) in the genome-wide association study analyses of thromboembolism in the imputed data including the sensitivity analyses. F2 = factor 2, F5 = factor V, adenovirus vector = ChAdOx1 nCoV-19/Vaxzevria®, mRNA = BNT162b2/Comirnaty® and mRNA-1273/Spikevax®, MAF = minor allele frequency, N = number of cases and controls, OR = odds ratio, CI = confidence interval.

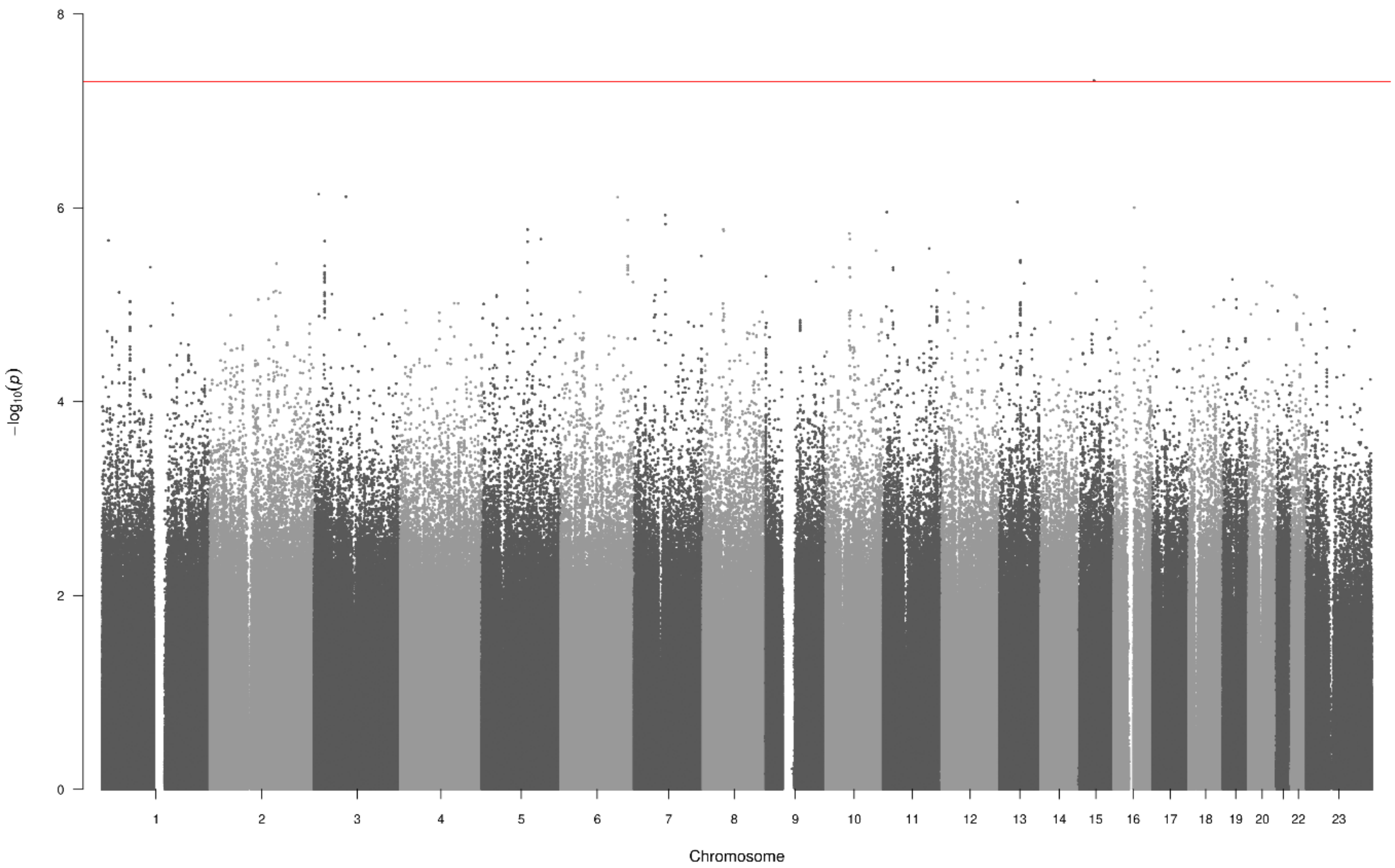

Supporting Figure S5. Manhattan plot of the genome-wide association study sensitivity analysis of thromboembolism in the imputed data (n = 185). The red line denotes the genome-wide significance level $P < 5{\times}10^{-8}$. Adjusted by sex and genetic principal components 1-4. One SNP passed the significance threshold, rs185809943 (Supporting Table S5).

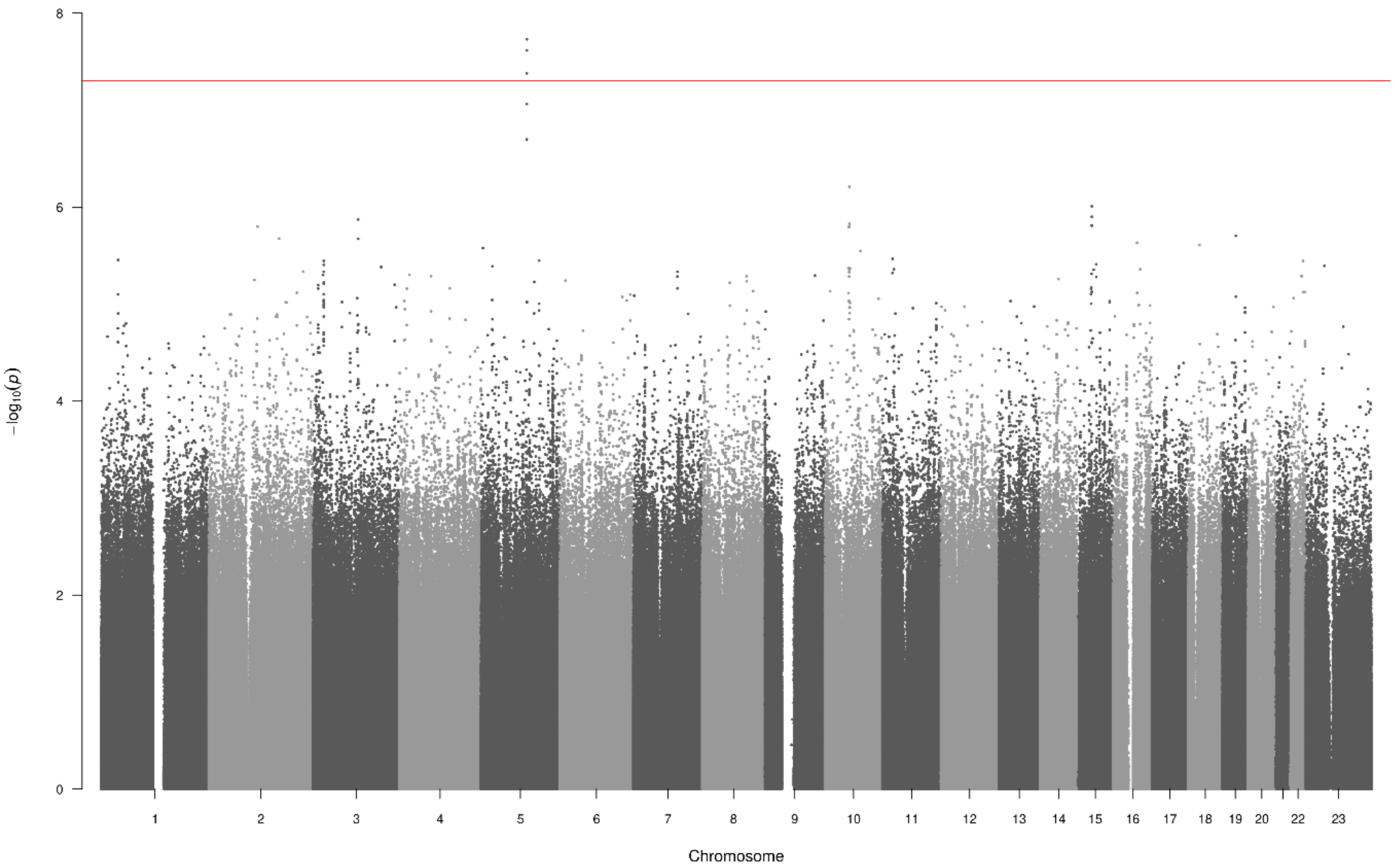

Supporting Figure S6. Manhattan plot of the mRNA subgroup genome-wide association study sensitivity analysis of thromboembolism in the imputed data (n = 130). The red line denotes the genome-wide significance level $P < 5\times10^{-8}$. Adjusted by sex and genetic principal components 1-4. Six SNPs passed the significance threshold; rs1272761321, rs12515306, rs112708383, rs113884741, rs76648032, and rs77838176 (Supporting Table S6).

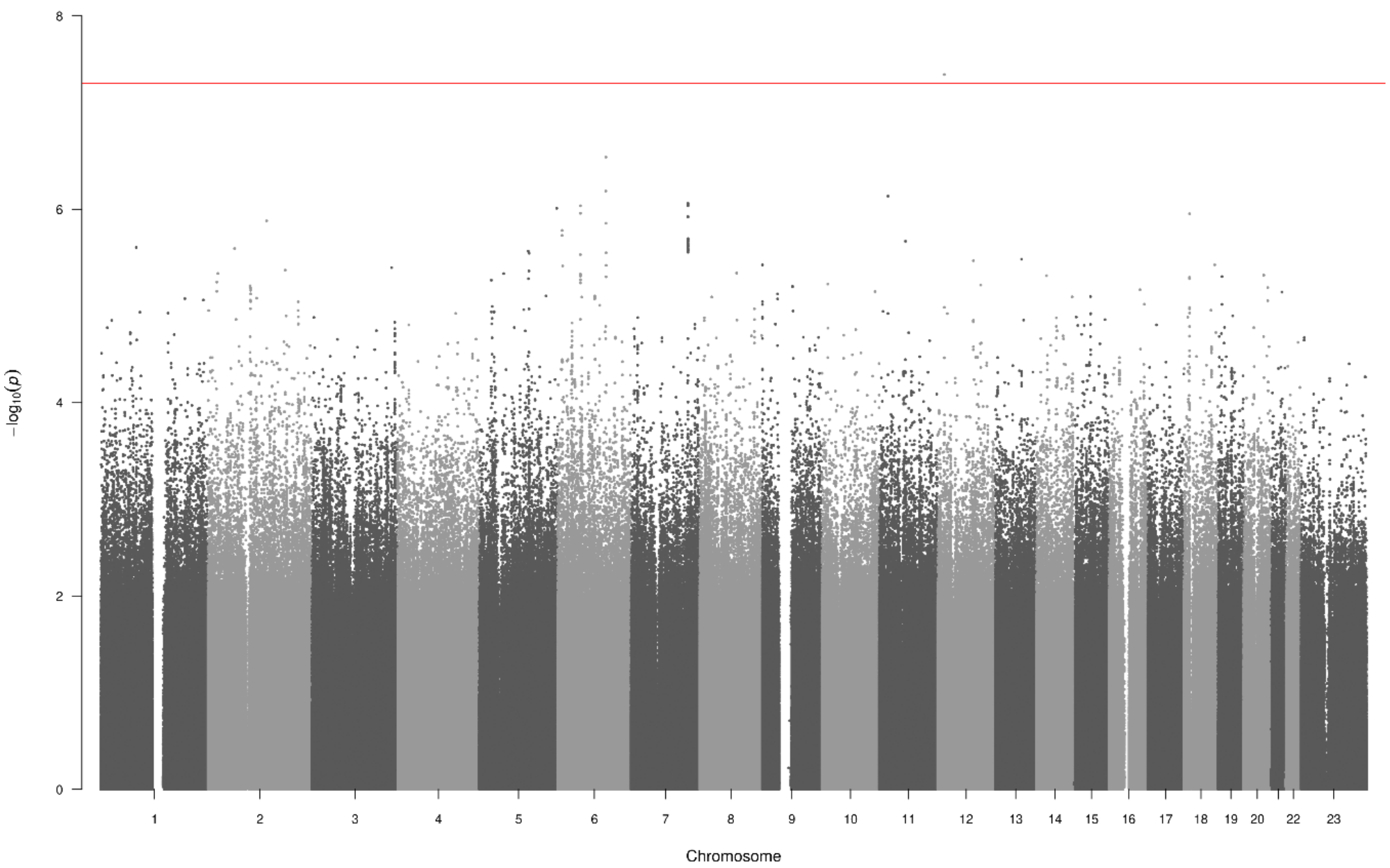

Supporting Figure S7. Manhattan plot of the adenovirus vector vaccine subgroup genome-wide association study sensitivity analysis of thromboembolism in the imputed data (n = 55).
The red line denotes the genome-wide significance level $P < 5\times10^{-8}$. Adjusted by sex and genetic principal components 1-4. One SNP passed the significance threshold, rs561929057 (Table S7).

Supporting Table S1. Candidate gene results for the gene set analyses for all cases of thromboembolism (n = 201) vs all controls (n = 4,891)

Region	CHR	max MAF	P value SKAT-O	P value Burden	P value SKAT	BETA Burden	SE Burden	MAC case	MAC control	Number rare	Number ultra rare
F5	1	0.01	0.4363	0.8349	0.2806	-0.003936	0.01888	5	115	5	13
F5	1	0.10	0.0413	0.0431	0.0329	0.017786	0.00879	159	3082	13	13
F5	1	0.20	0.0413	0.0431	0.0329	0.017786	0.00879	159	3082	13	13
F5	1	0.40	0.0420	0.0442	0.0329	0.017682	0.00879	514	13426	17	13
SERPINC1	1	0.01	0.8625	0.8883	0.7388	-0.004238	0.03018	2	54	2	2
SERPINC1	1	0.10	0.8625	0.8883	0.7388	-0.004238	0.03018	2	54	2	2
SERPINC1	1	0.20	0.8625	0.8883	0.7388	-0.004238	0.03018	2	54	2	2
SERPINC1	1	0.40	0.8625	0.8883	0.7388	-0.004238	0.03018	2	54	2	2
PROC	2	0.01	0.1209	0.1008	0.0803	0.036878	0.02248	7	97	3	1
PROC	2	0.10	0.1209	0.1008	0.0803	0.036878	0.02248	7	97	3	1
PROC	2	0.20	0.1209	0.1008	0.0803	0.036878	0.02248	7	97	3	1
PROC	2	0.40	0.1196	0.1008	0.0803	0.036883	0.02247	157	3489	4	1
HRG	3	0.01	1.0000	0.8276	0.8057	-0.003530	0.01621	8	195	4	8
HRG	3	0.10	1.0000	0.8276	0.8057	-0.003530	0.01621	8	195	4	8
HRG	3	0.20	1.0000	0.8386	0.8276	-0.003222	0.01582	123	3056	6	8
HRG	3	0.40	1.0000	0.8389	0.8276	-0.003217	0.01582	336	8287	8	8
KNG1	3	0.01	0.1788	0.1788	0.1788	0.103789	0.07719	1	7	0	3
KNG1	3	0.10	0.0735	0.1140	0.0685	-0.036442	0.02306	11	503	2	3
KNG1	3	0.20	0.0735	0.1140	0.0685	-0.036442	0.02306	11	503	2	3
KNG1	3	0.40	0.0735	0.1140	0.0685	-0.036442	0.02306	11	503	2	3
PROS1	3	0.01	1.0000	0.9474	0.9287	-0.002069	0.03138	2	52	2	2
PROS1	3	0.10	1.0000	0.9474	0.9287	-0.002069	0.03138	2	52	2	2
PROS1	3	0.20	1.0000	0.9474	0.9287	-0.002069	0.03138	2	52	2	2
PROS1	3	0.40	1.0000	0.9474	0.9287	-0.002069	0.03138	2	52	2	2
KLKB1	4	0.01	0.3743	0.6549	0.2720	0.010329	0.02311	5	108	1	6
KLKB1	4	0.10	0.3496	0.9469	0.2409	-0.001309	0.01966	33	993	2	6

Region	CHR	max MAF	P value SKAT-O	P value Burden	P value SKAT	BETA Burden	SE Burden	MAC case	MAC control	Number rare	Number ultra rare
KLKB1	4	0.20	0.3579	0.9720	0.2459	0.000437	0.01243	86	2181	3	6
KLKB1	4	0.40	0.3579	0.9721	0.2459	0.000433	0.01236	213	5355	4	6
PLG	6	0.01	0.8122	0.6165	0.9882	-0.007348	0.01467	9	224	7	4
PLG	6	0.10	0.4333	0.2735	0.9452	-0.012056	0.01101	16	495	9	4
PLG	6	0.20	0.4333	0.2735	0.9452	-0.012056	0.01101	16	495	9	4
PLG	6	0.40	0.4336	0.2736	0.9452	-0.012052	0.01101	135	3296	10	4
ADAMTS13	9	0.01	0.3529	0.4614	0.2269	-0.012582	0.01708	5	174	4	13
ADAMTS13	9	0.10	0.4867	0.9417	0.3205	-0.000936	0.01281	32	801	6	13
ADAMTS13	9	0.20	0.5050	0.9581	0.3344	-0.000680	0.01294	176	4425	9	13
ADAMTS13	9	0.40	0.5050	0.9581	0.3344	-0.000680	0.01294	176	4425	9	13
F2	11	0.01	0.3450	0.2620	0.3409	-0.041834	0.03729	0	34	1	2
F2	11	0.10	0.3450	0.2620	0.3409	-0.041834	0.03729	0	34	1	2
F2	11	0.20	0.4611	0.3475	0.3526	-0.034285	0.03649	61	1340	2	2
F2	11	0.40	0.4611	0.3475	0.3526	-0.034285	0.03649	61	1340	2	2
THBD	20	0.01	1.0000	0.8700	0.9639	-0.006501	0.03971	1	31	1	3
THBD	20	0.10	1.0000	0.8700	0.9639	-0.006501	0.03971	1	31	1	3
THBD	20	0.20	1.0000	0.8700	0.9639	-0.006501	0.03971	1	31	1	3
THBD	20	0.40	1.0000	0.8741	0.9646	-0.006292	0.03971	95	2270	2	3
SERPIND1	22	0.01	0.4501	0.5780	0.3184	-0.014176	0.02548	2	76	2	2
SERPIND1	22	0.10	0.4501	0.5780	0.3184	-0.014176	0.02548	2	76	2	2
SERPIND1	22	0.20	0.4501	0.5780	0.3184	-0.014176	0.02548	2	76	2	2
SERPIND1	22	0.40	0.4501	0.5780	0.3184	-0.014176	0.02548	2	76	2	2
PIGA	X	0.10	0.2402	0.2402	0.2402	0.017518	0.01491	19	315	1	0
PIGA	X	0.20	0.2402	0.2402	0.2402	0.017518	0.01491	19	315	1	0
PIGA	X	0.40	0.2402	0.2402	0.2402	0.017518	0.01491	19	315	1	0

CHR = chromosome, MAF = minor allele frequency, max MAF = this is the cutoff for the maximum MAF of the variants used in the gene set analysis, SKAT = sequence kernel association test, BETA = the beta coefficient is the degree of change in the outcome variable for every unit of change in the predictor variable, SE = standard error, MAC = minor allele count, Number rare = the number of variants with MAC above 10 in total, Number ultra rare = the number of variants with MAC below or equal to 10. These are combined in the analysis. The P-value for significance was set to 0.0038, according to Bonferroni adjustment for the number of tested genes (n = 13).

Supporting Table S2. Top results for the candidate gene regions from the genome-wide association study analyses of the outcome thromboembolism (n = 201) vs all population controls (n = 4,891). Total number of SNPs with results 5,719

CHR	Position[a]	SNP	Alleles major/minor	MAF cases	MAF controls	OR [95% CI]	P	Gene
9	133443675	rs28446901	C/G	0.264	0.187	1.588 [1.26-2.001]	0.0001117098	ADAMTS13
1	169540781	rs115148599	T/A	0.007	0	27.88 [5.564-139.694]	0.0002259681	F5
9	133417268	rs1186182675	C/T	0.007	0	24.392 [4.849-122.702]	0.0003443891	REXO4
9	133440617	rs28645493	C/G	0.149	0.098	1.685 [1.267-2.241]	0.0004531892	ADAMTS13
3	93941576	rs533582239	C/T	0.005	0	142.197 [3.428-5899.259]	0.0005885275	PROS1
3	93959929	rs1031505353	C/T	0.005	0	142.197 [3.428-5899.259]	0.0005885275	PROS1
20	23038750	20:23038750	G/C	0.005	0	138.718 [3.322-5791.819]	0.0006305545	SSTR4
9	133465306	rs41302667	G/A	0.149	0.099	1.656 [1.244-2.204]	0.0007017459	CACFD1
2	127418062	rs181159030	T/G	0.007	0	18.016 [3.92-82.801]	0.0007588776	
2	127438467	rs566178860	A/G	0.007	0	18.016 [3.92-82.801]	0.0007588776	
9	133421954	9:133421954	TCTC/T	0.005	0	118.017 [2.846-4893.356]	0.0008400008	
9	133424478	rs142107133	C/T	0.005	0	118.017 [2.846-4893.356]	0.0008400008	ADAMTS13
9	133450853	rs36222279	G/C	0.149	0.1	1.642 [1.234-2.185]	0.0008467078	ADAMTS13
9	133446950	rs28602660	G/A	0.147	0.099	1.615 [1.212-2.152]	0.001313168	ADAMTS13
1	169569591	rs538429864	C/T	0.015	0.003	4.975 [2.098-11.798]	0.001635484	F5
6	160754622	rs561171617	G/T	0.005	0	46.169 [4.406-483.796]	0.001877214	
6	160756426	6:160756426	CTA/C	0.005	0	46.169 [4.406-483.796]	0.001877214	
1	169549811	rs6025	C/T	0.08	0.044	1.809 [1.252-2.615]	0.002240329	F5
6	160719265	rs143556831	T/C	0.017	0.004	4.546 [1.92-10.764]	0.002316427	PLG
9	133464832	rs3124764	C/T	0.204	0.15	1.483 [1.152-1.908]	0.002494936	CACFD1
6	160713273	rs528521448	A/T	0.017	0.004	4.431 [1.876-10.466]	0.002606504	PLG
6	160714676	rs4252103	A/G	0.017	0.004	4.431 [1.876-10.466]	0.002606504	PLG
6	160718118	rs142757312	G/A	0.017	0.004	4.431 [1.876-10.466]	0.002606504	PLG

CHR	Position[a]	SNP	Alleles major/minor	MAF cases	MAF controls	OR [95% CI]	P	Gene
2	127430837	rs1318879947	C/T	0.005	0	33.056 [4.525-241.487]	0.00276086	
9	133458632	rs4962153	G/A	0.204	0.151	1.475 [1.146-1.899]	0.00285617	ADAMTS13
2	127438848	rs6749002	T/G	0.381	0.453	0.73 [0.59-0.90]	0.002947767	
2	127437668	rs6754772	G/A	0.381	0.453	0.73 [0.59-0.90]	0.002956222	
2	127438712	rs201287747	G/GT	0.381	0.453	0.73 [0.59-0.90]	0.003071485	
9	133462696	rs78534112	G/A	0.062	0.035	1.939 [1.269-2.963]	0.003074343	CACFD1
6	160705709	rs148243035	T/C	0.015	0.004	4.388 [1.843-10.446]	0.003151587	PLG
9	133451821	rs3124766	G/A	0.204	0.152	1.467 [1.14-1.887]	0.003210111	ADAMTS13
9	133451294	rs781853781	C/G	0.007	0.001	9.643 [2.657-35.001]	0.003362299	ADAMTS13
9	133461126	rs739468	G/T	0.204	0.152	1.463 [1.137-1.882]	0.003432229	CACFD1
1	169545552	rs9332591	G/A	0.077	0.126	0.591 [0.408-0.855]	0.004154143	F5
6	160730936	rs73784945	T/C	0.02	0.005	3.646 [1.621-8.199]	0.004385198	PLG
4	186225655	rs6847184	C/T	0.005	0	25.389 [3.508-183.763]	0.004489688	
11	46736290	rs554009916	A/G	0.02	0.008	3.11 [1.539-6.286]	0.004557654	F2
1	169590542	rs3917849	G/A	0.005	0	17.451 [2.904-104.89]	0.004621841	SELP
1	169595392	rs3917821	G/A	0.005	0	17.451 [2.904-104.89]	0.004621841	SELP
1	169595783	rs3917816	G/A	0.005	0	17.451 [2.904-104.89]	0.004621841	SELP
11	46745151	rs77285996	A/G	0.087	0.054	1.699 [1.186-2.433]	0.004741632	CKAP5
6	160712527	rs4252091	A/G	0.017	0.005	3.581 [1.578-8.125]	0.005403955	PLG
2	127436706	rs184685615	T/A	0.017	0.006	3.347 [1.532-7.312]	0.006048331	
1	169503661	rs6670848	G/A	0.119	0.081	1.53 [1.124-2.083]	0.008034655	
1	169506814	rs10737547	G/A	0.119	0.081	1.53 [1.124-2.083]	0.008034655	
1	169508336	rs6687813	C/A	0.119	0.081	1.53 [1.124-2.083]	0.008034655	
1	169521163	rs6682179	C/T	0.119	0.081	1.53 [1.124-2.083]	0.00806111	F5
1	173916232	rs61827937	T/C	0.005	0	12.852 [2.382-69.357]	0.008098542	SERPINC1
2	127418286	rs1799808	C/T	0.294	0.355	0.745 [0.598-0.928]	0.008123873	
1	169531352	rs6427197	A/C	0.119	0.081	1.528 [1.123-2.08]	0.008233208	F5
1	169511883	rs6427194	A/T	0.119	0.081	1.528 [1.123-2.08]	0.008235897	

CHR	Position[a]	SNP	Alleles major/minor	MAF cases	MAF controls	OR [95% CI]	P	Gene
1	169511938	rs6427195	T/A	0.119	0.081	1.528 [1.123-2.08]	0.008235897	
2	127433384	rs6710535	C/G	0.294	0.355	0.745 [0.598-0.928]	0.008239664	
2	127427993	rs2069931	C/T	0.294	0.355	0.746 [0.599-0.93]	0.008667873	PROC
11	46739666	rs763121673	G/A	0.005	0.001	12.071 [2.35-62.013]	0.008993793	
6	160754406	rs148517610	G/A	0.015	0.004	3.777 [1.523-9.368]	0.009608093	
6	160761043	rs367956251	C/T	0.015	0.004	3.777 [1.523-9.368]	0.009608093	
6	160763577	rs12528526	A/G	0.015	0.004	3.777 [1.523-9.368]	0.009608093	
2	127417716	rs374441532	T/C	0.007	0.002	6.425 [1.925-21.445]	0.009705337	
1	169529596	rs6009	C/T	0.119	0.081	1.513 [1.112-2.058]	0.009773652	F5

CHR = chromosome, SNP = single nucleotide polymorphism, MAF = minor allele frequency, OR = odds ratio with lower and upper 95% confidence interval (CI).
[a] All results are reported in accordance with the Genome Reference Consortium human assembly GRCh38.

Supporting Table S3. Top 30 results of the genome-wide association analysis for all cases of thromboembolism (n=201) vs all controls (n=4,891).

CHR	Position[a]	SNP	Alleles major/ minor	MAF cases	MAF controls	OR	95% CI L95	95% CI U95	P	Gene
15	56795952	rs185809943	C/G	0.032	0.004	7.757	4.078	14.758	1.24E-07	TCF12-DT
11	23506639	rs139206312	C/T	0.025	0.002	11.438	5.348	24.461	1.33E-07	
1	163839927	rs192491595	T/C	0.022	0.002	12.648	5.669	28.218	2.29E-07	
6	156226552	rs9397913	C/T	0.197	0.107	2.03	1.572	2.621	2.71E-07	
20	42492599	rs773118790	C/T	0.012	0	54.763	10.946	273.99	4.67E-07	PTPRT
5	136255710	rs182161202	G/A	0.012	0	56.14	11.036	285.582	4.88E-07	TRPC7
11	23441005	rs183980853	G/A	0.02	0.002	13.469	5.666	32.017	7.06E-07	
6	156227435	rs4628111	T/A	0.172	0.09	2.055	1.568	2.694	8.05E-07	
5	145250269	rs181404992	G/T	0.022	0.002	10.897	4.881	24.332	8.49E-07	
6	156199025	rs9371972	G/T	0.172	0.091	2.041	1.556	2.676	1.02E-06	
3	10362867	3:10362867	CAT/C	0.012	0	45.059	10.362	195.949	1.07E-06	ATP2B2
6	156219074	rs9371413	A/G	0.172	0.091	2.037	1.553	2.671	1.08E-06	
6	156204903	rs9384436	G/T	0.172	0.091	2.037	1.553	2.671	1.08E-06	
5	145257639	rs142363619	T/C	0.022	0.002	10.491	4.726	23.289	1.09E-06	
6	132681216	rs188311958	C/T	0.012	0	37.421	9.894	141.529	1.13E-06	VNN1
6	156206254	rs73581351	T/C	0.172	0.091	2.033	1.55	2.667	1.15E-06	
3	73371372	rs140951126	C/G	0.01	0	95.263	12.127	748.338	1.19E-06	
5	94266953	rs144312171	A/G	0.047	0.013	4.125	2.501	6.805	1.24E-06	KIAA0825
6	156223407	rs9384439	A/G	0.172	0.092	2.024	1.543	2.653	1.30E-06	
6	156224345	rs4435954	G/C	0.172	0.092	2.024	1.543	2.653	1.30E-06	
5	179316026	rs569659871	C/T	0.015	0.001	24.124	7.55	77.078	1.33E-06	ADAMTS2
6	52996176	rs36012838	G/T	0.134	0.069	2.185	1.622	2.944	1.51E-06	
6	52996235	rs34450404	C/G	0.134	0.069	2.185	1.622	2.944	1.51E-06	
6	52996697	rs1561990372	AT/A	0.134	0.069	2.185	1.622	2.944	1.51E-06	
6	52990900	rs72932939	C/T	0.134	0.069	2.184	1.621	2.942	1.54E-06	GSTA4

CHR	Position[a]	SNP	Alleles major/ minor	MAF cases	MAF controls	OR	95% CI L95	95% CI U95	P	Gene
10	18671000	rs34460788	A/G	0.027	0.004	7.143	3.628	14.065	1.54E-06	ARL5B
16	47793850	rs191137652	G/A	0.01	0	90.289	11.387	715.917	1.59E-06	
5	133716848	rs73789726	G/A	0.015	0.001	22.47	7.086	71.253	1.73E-06	
11	8718178	rs182033994	T/C	0.012	0	33.951	8.985	128.293	1.80E-06	DENND2B
6	53003599	rs113189631	C/T	0.132	0.069	2.174	1.61	2.938	2.11E-06	CILK1

CHR = chromosome, SNP = single nucleotide polymorphism, MAF = minor allele frequency, OR = odds ratio with lower and upper 95% confidence interval (CI). The P-value for significance was set to the conventional genome-wide significance threshold $P < 5 \times 10^{-8}$. Total number of SNPs with results were 24,382,386.

[a] All results are reported in accordance with the Genome Reference Consortium human assembly GRCh38.

Supporting Table S4. Post-hoc: Human Leukocyte Antigen (HLA) imputation results. Top results for the HLA analyses of the outcome thromboembolism (n = 201) vs all population controls (n = 4,891).

Total number of HLA variants with results 181.

CHR	Position[a]	HLA haplotype	Haplotype Frequency Cases	Haplotype Frequency Controls	OR	95% CI L95	95% CI U95	P
6	33043716	HLA_DPB1*06:01	0.032	0.015	2.356	1.326	4.186	0.005544349
6	31321785	HLA_B*56:01	0.022	0.008	2.81	1.392	5.675	0.006899798
6	29910339	HLA_A*68:01	0.067	0.042	1.652	1.097	2.486	0.01947902
6	31321754	HLA_B*47:01	0.012	0.005	3.144	1.256	7.872	0.0224791
6	32546560	HLA_DRB1*04:05	0.01	0.003	3.656	1.326	10.081	0.02269338
6	31321788	HLA_B*57:01	0.01	0.027	0.382	0.15	0.974	0.02492639
6	31321690	HLA_B*27:02	0.007	0.002	4.413	1.353	14.395	0.02679213
6	31236554	HLA_C*07:04	0.032	0.02	1.846	1.048	3.253	0.04317005
6	33043706	HLA_DPB1*02:01	0.107	0.137	0.722	0.521	1	0.04363699
6	31321778	HLA_B*53:01	0.005	0.001	13.779	0.967	196.387	0.05297698
6	31321716	HLA_B*39:01	0	0.008	0.344	0.109	1.081	0.06765179
6	32546563	HLA_DRB1*04:08	0.012	0.005	3.456	0.911	13.12	0.06839278
6	29910286	HLA_A*24:02	0.057	0.082	0.714	0.494	1.031	0.0720234
6	32546572	HLA_DRB1*08:01	0.065	0.042	1.551	0.961	2.504	0.07229361
6	31321698	HLA_B*35:02	0	0.002	0.252	0.053	1.192	0.08205478
6	32546587	HLA_DRB1*11:04	0.002	0.006	0.331	0.095	1.153	0.08246117
6	31236530	HLA_C*02:02	0.077	0.058	1.459	0.949	2.243	0.08486769
6	31321798	HLA_B*67:01	0.002	0	154.764	0.49	48890.677	0.08597828
6	32605194	HLA_DQA1*04:01	0.067	0.045	1.488	0.934	2.372	0.09444983
6	32546549	HLA_DRB1*01:02	0.002	0.005	0.343	0.098	1.204	0.0948173
6	33043714	HLA_DPB1*05:01	0.035	0.022	1.747	0.896	3.407	0.1014483
6	32627259	HLA_DQB1*04:02	0.067	0.045	1.473	0.926	2.343	0.1015739
6	32627263	HLA_DQB1*05:03	0.012	0.023	0.577	0.296	1.125	0.1065003
6	33043740	HLA_DPB1*16:01	0.002	0.01	0.438	0.161	1.195	0.1070066
6	31321750	HLA_B*45:01	0.01	0.004	3.286	0.755	14.297	0.1128483

CHR	Position[a]	HLA haplotype	Haplotype Frequency Cases	Haplotype Frequency Controls	OR	95% CI L95	95% CI U95	P
6	32546608	HLA_DRB1*14:01	0.012	0.023	0.582	0.297	1.138	0.113768
6	31321666	HLA_B*15:03	0.002	0	25.632	0.44	1492.173	0.1177243
6	31321693	HLA_B*27:05	0.08	0.063	1.382	0.911	2.098	0.1280638
6	29910278	HLA_A*11:01	0.067	0.052	1.416	0.897	2.235	0.1353021
6	32546562	HLA_DRB1*04:07	0	0.006	0.348	0.087	1.392	0.1355916
6	32546550	HLA_DRB1*01:03	0	0.006	0.348	0.085	1.422	0.1416082
6	29910309	HLA_A*30:02	0	0.005	0.348	0.085	1.424	0.1421299
6	31236566	HLA_C*12:03	0.017	0.027	0.625	0.33	1.184	0.1497798
6	31236561	HLA_C*08:02	0.01	0.019	0.592	0.288	1.215	0.1531547
6	32546548	HLA_DRB1*01:01	0.117	0.094	1.273	0.908	1.784	0.1609558
6	31236579	HLA_C*16:02	0.002	0.001	14.244	0.329	616.681	0.1670568
6	31236527	HLA_C*01:02	0.052	0.038	1.44	0.858	2.417	0.1672224
6	29910252	HLA_A*02:01	0.313	0.35	0.863	0.698	1.066	0.1716449
6	29910311	HLA_A*30:04	0.005	0.002	4.704	0.504	43.867	0.1740283
6	29910299	HLA_A*26:01	0.012	0.022	0.626	0.313	1.253	0.1861743
6	29910320	HLA_A*32:01	0.02	0.033	0.681	0.383	1.21	0.1900387
6	31236536	HLA_C*03:04	0.122	0.147	0.828	0.623	1.101	0.1934861
6	29910273	HLA_A*03:01	0.192	0.165	1.195	0.913	1.565	0.1944784
6	31236582	HLA_C*17:01	0	0.004	0.349	0.071	1.715	0.1950716
6	29910323	HLA_A*33:01	0	0.004	0.366	0.08	1.674	0.1951853
6	32546559	HLA_DRB1*04:04	0.027	0.045	0.717	0.43	1.198	0.2040858
6	32546593	HLA_DRB1*12:01	0.032	0.023	1.507	0.783	2.903	0.219628
6	31236535	HLA_C*03:03	0.087	0.072	1.272	0.862	1.877	0.2247101
6	31321661	HLA_B*14:02	0.007	0.014	0.609	0.26	1.424	0.252401
6	32546558	HLA_DRB1*04:03	0.01	0.005	2.22	0.566	8.702	0.2524195
6	31321658	HLA_B*13:02	0.005	0.01	0.568	0.215	1.501	0.2540169
6	32546556	HLA_DRB1*04:01	0.104	0.132	0.844	0.623	1.144	0.2744439

CHR	Position[a]	HLA haplotype	Haplotype Frequency Cases	Haplotype Frequency Controls	OR	95% CI L95	95% CI U95	P
6	29910315	HLA_A*31:01	0.037	0.03	1.394	0.765	2.539	0.2780969
6	31321737	HLA_B*41:02	0	0.003	0.351	0.052	2.354	0.2809562
6	32546598	HLA_DRB1*13:03	0.002	0.007	0.513	0.15	1.756	0.2877862
6	32627270	HLA_DQB1*06:04	0.045	0.056	0.79	0.511	1.223	0.2910161
6	33043711	HLA_DPB1*04:01	0.475	0.456	1.112	0.908	1.362	0.3032645
6	31321684	HLA_B*18:01	0.03	0.041	0.77	0.459	1.29	0.3209721
6	29910287	HLA_A*24:03	0.002	0.001	5.024	0.206	122.402	0.3217956
6	32627268	HLA_DQB1*06:02	0.167	0.155	1.145	0.866	1.514	0.3433672

CHR = chromosome, HLA = human leukocyte antigen, MAF = minor allele frequency, OR = odds ratio with lower and upper 95% confidence interval (CI).
[a] All results are reported in accordance with the Genome Reference Consortium human assembly GRCh38.

Supporting Table S5. Top results of the genome-wide association sensitivity analysis for cases of thromboembolism (n=185) vs all controls (n=4,891).

CHR	Position[a]	SNP	Alleles major/ minor	MAF cases	MAF controls	OR	95% CI L95	95% CI U95	P	Gene
15	56795952	rs185809943	C/G	0.035	0.004	8.496	4.452	16.213	0.00000004867042*	TCF12-DT
3	10362867	3:10362867	CAT/C	0.014	0	49.38	11.299	215.804	0.0000007224618	ATP2B2
3	73371372	rs140951126	C/G	0.011	0	109.512	13.669	877.399	0.0000007676763	
6	132681216	rs188311958	C/T	0.014	0	40.619	10.705	154.129	0.0000007769774	VNN1
13	60754679	rs12874885	G/A	0.241	0.142	1.906	1.49	2.438	0.0000008686432	LINC00378
16	47793850	rs191137652	G/A	0.011	0	104.285	12.953	839.581	0.0000009940646	
11	8718178	rs182033994	T/C	0.014	0	37.87	9.961	143.976	0.000001108034	DENND2B
7	72130419	rs143552985	A/C	0.027	0.003	8.659	4.172	17.972	0.000001186793	CALN1
7	72196109	7:72196109	TTTTTC/T	0.027	0.003	8.659	4.172	17.972	0.000001186793	CALN1
6	156226552	rs9397913	C/T	0.195	0.107	1.997	1.53	2.608	0.000001336534	
7	72589177	rs185871070	T/C	0.027	0.003	8.449	4.069	17.544	0.000001472487	TYW1B
8	47320747	rs551341375	C/G	0.019	0.002	14.588	5.873	36.234	0.000001671477	SPIDR
8	47749346	rs572240620	G/A	0.019	0.002	14.588	5.873	36.234	0.000001671477	
8	48087493	rs147063012	C/T	0.019	0.002	14.588	5.873	36.234	0.000001671477	
5	105496459	rs1272761321	G/GA	0.038	0.008	5.449	3.015	9.846	0.000001677218	
8	48452304	rs552156224	T/A	0.019	0.002	14.526	5.846	36.094	0.000001725119	
10	55732219	rs11004991	C/T	0.014	0	77.382	9.982	599.848	0.000001838286	
5	136121566	rs369727624	T/A	0.011	0	217.031	7.926	5942.89	0.000002103072	
10	57152411	rs185380891	A/C	0.014	0	39.555	8.97	174.438	0.00000210745	
1	15109157	rs566268483	G/A	0.022	0.002	11.3	4.89	26.109	0.000002170093	KAZN

CHR = chromosome, SNP = single nucleotide polymorphism, MAF = minor allele frequency, OR = odds ratio with lower and upper 95% confidence interval (CI).
*The P-value for significance was set to the conventional genome-wide significance threshold $P < 5\times10^{-8}$. Total number of SNPs with results were 24,376,912.
[a] All results are reported in accordance with the Genome Reference Consortium human assembly GRCh38.

Supporting Table S6. Top results of the mRNA subgroup genome-wide association sensitivity analysis for cases of thromboembolism (n=130) vs all controls (n=4,891).

CHR	Position[a]	SNP	Alleles major/ minor	MAF cases	MAF controls	OR	95% CI L95	95% CI U95	P	Gene
5	105496459	rs1272761321	G/GA	0.054	0.008	8.267	4.527	15.096	0.00000001859627*	
5	105319531	rs12515306	C/T	0.054	0.008	8.06	4.423	14.689	0.00000002425545*	
5	105341261	rs112708383	T/C	0.054	0.008	8.06	4.423	14.689	0.00000002425545*	
5	105424323	rs113884741	A/C	0.054	0.008	8.059	4.422	14.688	0.00000002426255*	
5	105428735	rs76648032	T/A	0.054	0.008	8.059	4.422	14.688	0.00000002426255*	
5	105254262	rs77838176	T/C	0.054	0.009	7.672	4.218	13.955	0.00000004170899*	
5	105107301	rs76738801	T/A	0.054	0.009	7.193	3.959	13.068	0.00000008661955	
5	105221352	rs111449317	A/T	0.046	0.007	8.085	4.242	15.407	0.0000002009483	
10	57152411	rs185380891	A/C	0.019	0	54.932	12.231	246.699	0.0000006185623	
15	52778931	rs545932069	T/C	0.035	0.005	9.935	4.749	20.784	0.000000981911	ONECUT1
15	52772022	rs181574377	C/G	0.035	0.005	9.616	4.604	20.084	0.000001255777	ONECUT1
3	103518050	rs73852115	G/A	0.223	0.119	2.223	1.637	3.019	0.000001340258	
10	57312106	rs555961244	G/A	0.019	0	41.141	10.297	164.377	0.000001481085	
10	57323377	rs531932856	C/T	0.019	0	41.141	10.297	164.377	0.000001481085	
10	57381601	rs79831210	T/G	0.019	0	41.141	10.297	164.377	0.000001481085	
10	57383820	rs11005776	A/C	0.019	0	41.141	10.297	164.377	0.000001481085	
10	57390861	rs546117009	T/G	0.019	0	41.141	10.297	164.377	0.000001481085	
10	57405648	rs186500804	G/A	0.019	0	41.141	10.297	164.377	0.000001481085	
15	52756687	rs191654800	C/G	0.035	0.005	9.356	4.487	19.508	0.00000154158	ONECUT1
15	52732055	rs149758507	C/G	0.035	0.005	9.343	4.481	19.48	0.000001556588	

CHR = chromosome, SNP = single nucleotide polymorphism, MAF = minor allele frequency, OR = odds ratio with lower and upper 95% confidence interval (CI).
*The P-value for significance was set to the conventional genome-wide significance threshold $P < 5\times10^{-8}$. Total number of SNPs with results were 24,350,859.
[a] All results are reported in accordance with the Genome Reference Consortium human assembly GRCh38.

Supporting Table S7. Top results of the adenovirus vector vaccine subgroup genome-wide association sensitivity analysis for cases of thromboembolism (n=55) vs all controls (n=4,891).

CHR	Position[a]	SNP	Alleles major/ minor	MAF cases	MAF controls	OR	95% CI L95	95% CI U95	P	Gene
12	16140824	rs561929057	A/G	0.036	0	175.072	32.048	956.403	0.00000004045567*	
6	112271862	rs190629176	T/C	0.055	0.003	25.364	10.073	63.866	0.0000002891279	LAMA4-AS1
6	112142407	rs149446083	T/C	0.064	0.005	16.01	6.977	36.737	0.000000649196	LAMA4
11	20207822	rs185175456	C/A	0.064	0.005	15.708	6.847	36.039	0.0000007320343	
11	20208099	rs185002698	C/A	0.064	0.005	15.708	6.847	36.039	0.0000007320343	
7	131992569	7:131992569	AG/A	0.464	0.245	2.702	1.856	3.933	0.0000008685793	
7	131989394	rs9656406	G/A	0.464	0.245	2.699	1.854	3.93	0.0000008986155	
7	131990532	rs4288320	T/G	0.464	0.245	2.698	1.853	3.928	0.0000009203435	
6	53003599	rs113189631	C/T	0.209	0.069	3.798	2.379	6.065	0.0000009218708	CILK1
6	53014789	rs72932963	C/G	0.209	0.069	3.798	2.379	6.065	0.0000009218708	CILK1
5	179316026	rs569659871	C/T	0.036	0.001	61.388	16.734	225.201	0.0000009771144	ADAMTS2
6	52990900	rs72932939	C/T	0.209	0.069	3.741	2.347	5.963	0.000001099482	GSTA4
6	52996176	rs36012838	G/T	0.209	0.069	3.741	2.347	5.962	0.000001101821	
6	52996235	rs34450404	C/G	0.209	0.069	3.741	2.347	5.962	0.000001101821	
6	52996697	rs1561990372	AT/A	0.209	0.069	3.741	2.347	5.962	0.000001101821	
18	13565729	rs12458407	T/C	0.418	0.206	2.718	1.862	3.966	0.000001115192	LDLRAD4
7	131984616	rs1123431	C/G	0.464	0.246	2.666	1.831	3.881	0.000001191028	
7	131988475	rs925631	T/G	0.464	0.246	2.665	1.83	3.881	0.000001202653	
2	136322317	rs192659942	G/A	0.036	0.001	55.085	15.666	193.686	0.000001319181	
6	112248807	rs1219860324	G/GA	0.064	0.005	14.208	6.214	32.486	0.000001396767	LAMA4 | LAMA4-AS1

CHR = chromosome, SNP = single nucleotide polymorphism, MAF = minor allele frequency, OR = odds ratio with lower and upper 95% confidence interval (CI).
*The P-value for significance was set to the conventional genome-wide significance threshold $P < 5\times10^{-8}$. Total number of SNPs with results were 22,278,211.
[a] All results are reported in accordance with the Genome Reference Consortium human assembly GRCh38.
